# GLAD Based Advanced Nanostructures for Diversified Biosensing Applications: Recent Progress

**DOI:** 10.3390/bios12121115

**Published:** 2022-12-02

**Authors:** Sarjana Yadav, Sneha Senapati, Samir Kumar, Shashank K. Gahlaut, Jitendra P. Singh

**Affiliations:** 1 Department of Physics, Indian Institute of Technology Delhi, Hauz Khas, New Delhi 110016, India; 2 School of Interdisciplinary Research, Indian Institute of Technology Delhi, Hauz Khas, New Delhi 110016, India; 3 Department of Electronics and Information Engineering, Korea University, Sejong 30019, Republic of Korea

**Keywords:** glancing angle deposition (GLAD), biosensors, biomedical detection, GLAD substrates, plasmonics

## Abstract

Glancing angle deposition (GLAD) is a technique for the fabrication of sculpted micro- and nanostructures under the conditions of oblique vapor flux incident and limited adatom diffusion. GLAD-based nanostructures are emerging platforms with broad sensing applications due to their high sensitivity, enhanced optical and catalytic properties, periodicity, and controlled morphology. GLAD-fabricated nanochips and substrates for chemical and biosensing applications are replacing conventionally used nanomaterials due to their broad scope, ease of fabrication, controlled growth parameters, and hence, sensing abilities. This review focuses on recent advances in the diverse nanostructures fabricated via GLAD and their applications in the biomedical field. The effects of morphology and deposition conditions on GLAD structures, their biosensing capability, and the use of these nanostructures for various biosensing applications such as surface plasmon resonance (SPR), fluorescence, surface-enhanced Raman spectroscopy (SERS), and colorimetric- and wettability-based bio-detection will be discussed in detail. GLAD has also found diverse applications in the case of molecular imaging techniques such as fluorescence, super-resolution, and photoacoustic imaging. In addition, some in vivo applications, such as drug delivery, have been discussed. Furthermore, we will also provide an overview of the status of GLAD technology as well as future challenges associated with GLAD-based nanostructures in the mentioned areas.

## 1. Introduction

Biosensors have emerged in recent decades as a quintessential tool in the fields of healthcare, bioscience, the food industry, the chemical and biomedical industries, defense and security, and environmental monitoring [1]. Any system or chip consisting of a surface that is capable of binding to a specific biological substance that recognizes it, together with the ability to translate the biophysical or chemical reaction into a quantifiable signal, can be referred to as a biosensor [1]. The biosensor recognizing element may be nucleic acids, proteins, carbohydrates, lipids, antibodies, or an entire cell. These elements may be attached or immobilized on the sensor using various methods such as adsorption, bond formation, encapsulation, or entrapment [2]. Selectivity, specificity, reproducibility, stability, and sensitivity are various characteristics of a typical biosensor. Recently, the scientific community has witnessed vast development in optical, chemical, and physical transducers along with a specific affinity for biochemical interaction. This has led to vast progress in the development of biosensors in various fields. The most widely and extensively used biosensors are those that are based on nano-regimes due to their low detection limits, high stability, easy operation, low cost, short response time, and low power requirement [2]. Metallic-based nanostructures, therefore, possess specific unique features that ensure high sensitivity of the biosensors compared to other planar sensor configurations due to their nanoscale dimension and periodicity, thus ensuring strong binding of the target analyte and increased sensing properties [3].

The rapid advancement in the field of biosensing utilizing plasmonics has led to an extensive study of metallic nanostructures and their sensing applications [4,5,6]. One such emerging tool for fabricating metallic nanostructures is the glancing angle deposition (GLAD) technique [7]. It is a physical vapor deposition (PVD) process in which the gaseous flow of depositing atoms impinges on the substrate in a vacuum at an oblique angle such that the deposited film results in a columnar morphology [7,8] as a result of the shadowing effect. The tilting of the as-deposited column is controlled by the angle of incidence. The morphology of the columnar structure can be modified using a mobile substrate whose position can be managed during deposition [9,10]. The GLAD technique is considered better than the other chemical growth methods in terms of ease of fabrication of nanostructures due to controlled size, shape and spacing of columns in single step [11] Additionally, the nanostructures grown by this method possess high surface area to volume ratio [12], high porosity [13], crystallinity, and uniformity [11,14] making it the most suitable method for sensing applications. These structure has been widely investigated in designing different sensor devices [15], energy devices [16], and optical devices [17]. The GLAD technique has been extensively studied and developed over the past 15 years, but there is still much to explore.

The last few decades have witnessed a high increment in gold and silver-based nanostructures for catalytic [18] and biomedical applications such as immunotherapy, nanomedicine, and in vitro diagnostics [19,20,21,22]. Silver- and gold-based nanostructures are most widely used for sensing applications due to their high plasmonic properties [23,24,25], particularly their extinction band lying in the visible region [26] and low dispersion losses. Silver has a strong bactericidal effect due to the interaction of Ag ions with the thiol group and antimicrobial activity against microbes [27,28]. Gold has an excellent affinity for binding to antibodies [29], and hence, is most suited for antigen–antibody surface plasmon resonance [30,31] (SPR)-based detection. Silver- and gold-based nanostructures are shown to be the preferred transducers for LSPR-based sensing [32], surface-enhanced Raman scattering (SERS)-based sensing [33], as well as fluorescence resonance-based energy transfer [34] (FRET) sensing [35]. The highest SERS enhancement obtained using Ag nanorods prepared using the GLAD technique is 10^9^ [36], so GLAD-based nanostructures combined with other techniques provides variations in metallic nanostructure shape, pattern, and period, extending the scope of their sensing abilities. The colorimetric- and wettability-based properties of these metallic nanostructures are found to be highly sensitive towards certain chemical and biological processes, revealing their use in biosensing detection [37,38,39]. The ongoing research in biosensor technology highlights an increase in the use of nanostructures and nanoparticles as a receptor or chip to enhance detection sensitivity and capability. Nanostructure-based biosensors or biochips fabricated using PVD methods exhibit advantages over conventionally prepared nanomaterials in terms of their high surface-to-volume ratio, fast response time, high versatility, multiple-fold increase in sensitivity, and better target-specific element recognition [40].

In this review, the biosensing and other imaging properties of GLAD-fabricated nanostructures are evaluated. We shall discuss the fabrication of these nanostructures with different types of substrate structures along with their implications in biosensing and other biomedical applications. The abilities of these nanostructures in various biosensing fields have been explored. A comparison of GLAD-based nanostructures with conventionally used nanoparticles and chips will be highlighted along with their advantages over the latter. There are various advantages of GLAD-based deposition over other chemical route depositions; for example, no precursor materials are needed in GLAD, which provides safety from toxic precursors and by-products [41]; atomic-level control of chemical composition and thickness can be afforded in this deposition [42]; and a higher temperature is not required and heat-sensitive substrates can be fabricated using this technique. GLAD is a green fabrication technique which produces very little pollution [43], promising high uniformity and repeatability in the structures [44,45,46].

## 2. Recent Strategies for Fabrication of Nanostructures Using the GLAD Technique

PVD methods such as sputtering, electron-beam vapor deposition, and thermal evaporation are most commonly used to fabricate thin films. When the vapor flux is normally incident over the substrate, thin-film formation takes place. However, if the angle of incidence is large with respect to the normal of the substrate surface (>70°), columnar structures are formed. This type of PVD deposition, carried out using either thermal evaporation or e-beam evaporation, is referred to as oblique-angle deposition (OAD) [3]. This extended OAD combined with azimuthal substrate rotation, giving various shapes and size of nano-columns, is referred as GLAD [14,47] or as dynamic oblique-angle deposition by some researchers [48]).

### 2.1. Setup Configuration of GLAD

**Figure 1:**
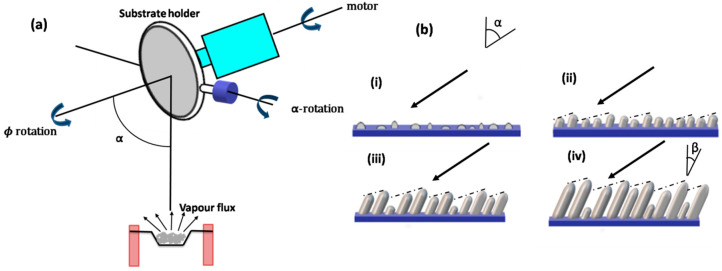
(**a**) Schematic of GLAD setup where α is the angle subtended by the incident vapor flux with respect to substrate normal and Φ is the azimuthal angle. (**b**) Mechanistic view of GLAD-based growth. (i) Incident vapor flux, shown by full arrows at an angle (α), resulting in random nucleation. (ii) Nucleus growth casting a nearby shadow, as shown by dotted lines. (iii) Column growth in progress, suppressing neighbors’ growth via shadowing. (iv) Full column growth at a certain specific angle (β) with respect to the normal.

Figure 1a shows the schematic of a GLAD setup where α is the angle subtended by the incident vapor with the substrate normal, called the deposition angle. Φ is the azimuthal angle that measures rotation about the substrate normal. The vapor flux falls on the substrate at a particular glancing angle (>700), initially leading to the formation of random nuclei, which is supported by the Volmer–Weber growth mechanism [49]. These further grow into isolated columns as a result of competition between shadowing and adatom diffusion due to larger nuclei and adatom surface diffusion. The columns lead to shadowing with size distribution and the screening of smaller neighboring nuclei from the coming flux. Therefore, the growth of the smaller nuclei is suppressed and ultimately ends due to complete shadowing [7]. This process of column extinction continues throughout the film’s growth.

More vapor flux deposition takes place as the nucleus growth continues developing into isolated columns. Finally, the isolated columns are formed, and are tilted towards the source of the vapor flux. β is the column tilt angle. This process is shown in Figure 1b. It must be noted that β is different from deposition angle α, and β = 0 for the columns parallel to the substrate. The relationship between angles α and β is given by the tangent rule, given by Niewenhuizen and Haanstra [50], as

(1)  tanα=2tanβ

when the deposition angle increases obliquely with respect to the normal, in which case, the tangent rule is not followed.

(2) $$\beta =\arcsin\left(\frac{1-\cos\alpha }{2}\right)$$

### 2.2. Effect of Various Growth Parameters

#### 2.2.1. Tilt Angle

As discussed earlier, the dependence of the tilt angle cannot be fully described in terms of angle of incidence by a single relationship. The general relationship is given by the tangent rule (Equation (1)), and Tait’s rule [51] (Equation (2)) is satisfied only under certain conditions and must be cross-verified for a specific material under the given conditions such as deposition rate, pressure, substrate type, and temperature. All these factors influence the tilt angle β. Nakhodkin and Shaldervan investigated the nature of films of different materials (Ge, Si, GeTe) as a function of tilt angle [52]. They found that most of the films are formed of crystallites inclined to the substrate due to obliquely incident vapor flux. They classified and correlated the structures based on material, cross-section, and tilt angle up to a thickness of 300 nm. Salazar et al. used GLAD-fabricated Cu electrodes at a 65° tilt angle to study their sensing ability for the detection of glucose in blood samples [53]. Most of the recent reports showing GLAD-based biosensing applications have employed tilt angles ranging from 65° to 85° with high sensitivity and reproducibility [53,54].

#### 2.2.2. Azimuthal Rotation (ϕ)

This represents the rotation of the substrate about the normal to the substrate. The azimuthal angle ϕ measures the rotation of the substrate, exploiting the growth direction steering the columnar structure along the growth path. The substrate position and the effect of azimuthal rotation on growth is depicted by α and ϕ and their variations. Control of both of these angles in a desirable manner during substrate rotation is the most crucial part of GLAD technology. If α and ϕ remain constant throughout the deposition, then it means that the substrate is stationary. α and ϕ may also change periodically at a given angle, but otherwise, may remain stationary [7]. There are six to nine basic GLAD structures that can be formed by different combinations of α and ϕ, which, along with their implications in biosensing, are discussed in detail in the next section. This variety of microstructures provides more enhanced properties that have a strong effect on the binding of biomolecules to the substrate and cause a change in the plasmonic properties of these substrates. Zhang et al. investigated the dependence of α and Φ on the biosensing ability of a Au nanostructured array fabricated over closely packed polystyrene balls and obtained the highest SPR and LSPR sensitivity for optimal values of α and Φ [55]. This regime as a function of tilt angle or azimuthal angle, due to the corresponding change in the substrate morphology and column growth, has not yet been fully explored and still has a long way to go.

### 2.3. GLAD-Based Nanostructures

The GLAD deposition technique can be used to create thin films of different morphology and sizes. As described in the previous section, the vapor flux falls on the substrate at a certain glancing angle (α > 70°), resulting in the formation of microstructures because of competition between the shadowing effect and adatom diffusion. The formation and categorization of various GLAD structures depend on the variation in α and φ during the deposition process.

The formation of the plane metallic film takes place when the substrate is held perpendicular to the vapor flux (α = 0°). However, this type of thin film is not very sensitive to biomolecule detection. Agrawal et al. has shown, using Rigorous coupled-wave analysis (RCWA) simulation, how nanohole array in a gold film can be employed for enhanced biosensing compared to planar gold film [56]. Some roughness over the surface of the nanostructure increases the sensitivity of biomolecules multiple-fold [57,58]. Slanted nanorods are the most commonly used GLAD structure for biosensing. These are formed when the substrate is held in the stationary position. α is taken to be greater than 70° (~86°). However, other desired micro- and nanostructures can be sculpted by varying the position of the substrate, and hence, the direction of the incoming vapor flux [59]. High sensitivity and plasmonic enhancement have been obtained using slanted nanorods with single arms and multiple arms [60]. Slanted nanorods have been utilized and studied widely in various biosensing applications using different materials (Ag, Si, Ni, TiO_2_) [60,61,62] due to their ease of fabrication, lower material requirement, lower complexity, and high optimized sensitivity [36].

**Figure 2:**
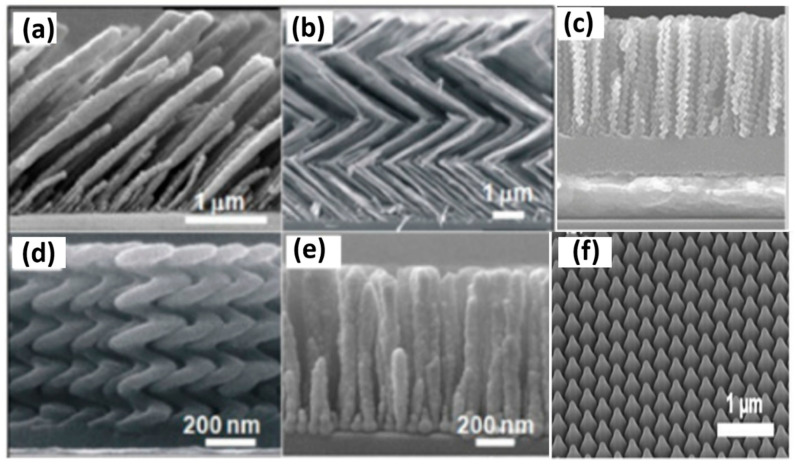
Different GLAD-structured thin films. (**a**) Slanted-post films; (**b**) zig-zag (chevron) films with 4 arms; (**c**) AgNR/SiO_2_ SCL/SiO_2_/PCL/Ag multilayer substrates; (**d**) helical film; (**e**) vertical post structure (reproduced with permission from [67]); (**f**) 45° tilted top view of Au mushrooms (reproduced with permission from [68]).

Chevron or zig-zag multi-armed nanorods are formed by a series of slanted nanorods. The substrate is initially held at a specific angle (α, φ) for the first arm, then, rotated by 180° (α, φ + π) for the second arm, and again by 180° (α, φ + 2π) for the third arm, etc.; that is, every time, the direction is rotated by 180° [7]. Square spirals can also be fabricated in the same way as chevrons with a rotation of 90°. The optimized slanted nanorods have shown enhanced biosensing and have been widely employed for SPR-, SERS-, colorimetric-, and wettability-based biosensing and detection. Sensitivity comparisons of various bioanalytes using various types of these GLAD-fabricated nanostructures have been performed by different groups [63]. Enhanced sensitivity, which can be employed for biological sensing using vertically standing nanoporous zig-zag or chevron nanorods providing hot spots with high SERS enhancement, has been shown by researchers [64,65]. Vertical nanorods are formed when the substrate is rotated at a constant rate during deposition. If the substrate is rotated at a constant slow rate, a helical columnar structure is formed with a constant pitch. Similarly, high and low morphology can be formed with a continuous change in φ while changing α in a discrete manner [66]. These structures form a Bragg stack and can be highly suitable for optical biosensing applications. Recently, Kumar et al. reviewed the fabrication and recent biochemical applications of various GLAD-fabricated substrates for SERS. They discussed, in depth, the advantages of these substrates and how these overcome the various limitations of conventional substrates [67], (Figure 2a–e). These GLAD-fabricated nanostructures have recently been employed in various biosensing detection settings. Recently, Kim et al. reported OAD-based LSPR substrates with a very high throughput by fabricating Au nanostructures (at 45°) over patterned sapphire substrates (PSS) [68] (Figure 2f). Very high LSPR sensitivity was obtained for various biomolecules and bioanalytes.

Hence, GLAD is a sophisticated technique used to design thin films with specific geometries. The geometries can be tailored in a controlled and desired manner to provide specific advantages for detection over conventional nanoparticles and nanofilms. The analyte binding sites, as well as the hotspots and scattering points (typically for optical sensing), can be increased in the desired way using this technique. The substrates can be fabricated in a controlled manner using multifunctional materials of different kinds by varying the substrate orientation and deposition conditions. The detailed bio-detection and sensitivity of various geometries and structures are yet to be explored in depth and could provide high scope in the future; they may provide certain specific binding sites for the proteins, amino acids, etc. present in biomolecules typically suited for SERS or fluorescence detection or antigen–antibody binding sites for SPR and LSPR.

## 3. Designing Substrates and Chips for Biosensing Applications

The sensitivity, binding, limit of detection, and other essential biosensing activities of a biosensor strongly depend on the substrate. However, there are only a few methods that can be used to develop uniform, reproducible, robust, stable, and cost-effective substrates. Recently, the GLAD technique has been employed to fabricate columnar thin-film substrates with high sensitivity [60,69]. Various studies have been conducted to study the sensing ability of the columnar Ag film [70,71]. Zhao et al. studied the s- and p-polarization absorbance spectra of Ag nanorod arrays with different heights and topologic shapes (cylindrical, needlelike, periodic, L, and Y shapes) in detail [72,73]. Additionally, the nanorods with complicated shapes, such as periodic, L, and Y shapes, had more hot spots for optical sensing applications than perfectly aligned nanorods. Thus, it is more suitable to observe the biosensing abilities of a small number of molecules on irregular nanorods than on a perfectly aligned nanorod. Recently, various types of GLAD-based nanostructures have been tested, optimized, and fabricated to increase the sensitivity of bioanalytes and expand their applications in the biomedical field. These substrates offer numerous advantages over the conventional GLAD-based substrates as they are more sensitive, durable, and flexible, and bind bioanalytes more effectively. The biosensing applications of the various newly fabricated substrates in the field of biomedicine and biosensing are discussed below.

### 3.1. Low-Temperature Biosensing Chips and Arrays

**Figure 3:**
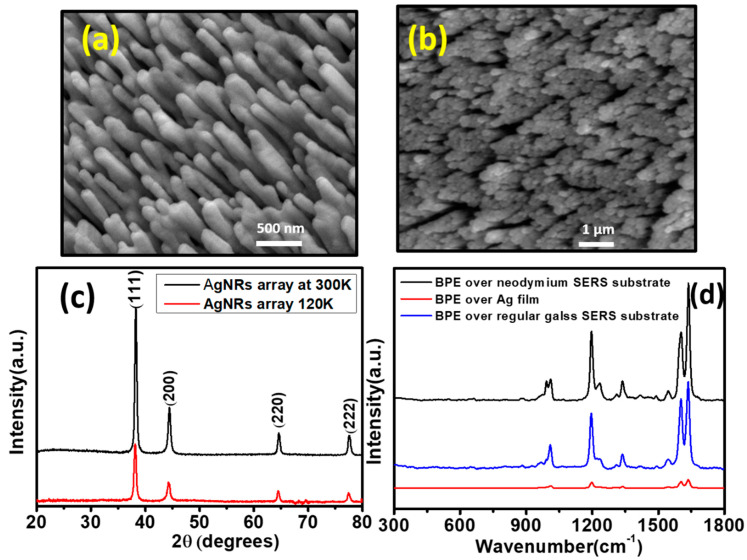
(**a**) SEM images of AgNRs over glass substrates at room temperature (300 K). (**b**) SEM images of AgNRs at 120 K deposited using GLAD technique. (**c**) XRD pattern for the two AgNR SERS substrates. (**d**) Comparison of the Raman and SERS spectra of BPE analyte over the three different substrates (c-SERS and M-SERS substrates, and 200 nm thin Ag film). Reproduced with permission from [63].

Aligned AgNR array films have been fabricated at low temperatures (LT; 140 K) and also at higher temperatures (HT; 373 K) [74,75]. The effects of temperature, from low to high, on the deposition of nanostructures and the morphology of films have already been discussed in the earlier section. Singh et al. deposited tilted AgNRs on glass slides at a substrate temperature of T = 140 K. The surface morphology changed drastically for the LT-deposited Ag nanostructured film. The aligned AgNR array SERS substrates deposited at a low-temperature oblique angle exhibited better SERS enhancement with good optical transmission compared to the standard room-temperature (RT)-deposited AgNR SERS substrates. The LT-deposited Ag nanostructured film was more porous, whereas the RT-deposited Ag nanostructured films appeared to be solid and rod-like. The nanorods with lengths of 1 μm, corresponding to a d = 2 μm QCM reading, deposited at room temperature showed the maximum spectral intensity, which is advantageous for optical (SERS, fluorescence, SPR) biosensing applications. However, for AgNR, the LT AgNR substrates, along with the maximum response, also provided substantial cost savings. A recent study by Yadav et al. demonstrated that magnetic surface-enhanced Raman spectroscopy (M-SERS) was effective in detecting hemozoin, a metabolic by-product of malarial parasites with paramagnetic properties. Using the GLAD technique, the SERS-active AgNRs were fabricated over neodymium substrates (0.3 T) kept at low temperature (120 K). Both hemozoin and hemoglobin were measured via a magnetic field-based SERS method on M-SERS substrates. In the absence of an external magnetic field, the intensity of the SERS signal was reportedly ten times greater than that of conventional SERS substrates. Figure 3a,b shows a SEM image of AgNR arrays fabricated over glass substrates at room temperature and at low temperature (120 K), respectively. Bunches of uniform nanorods were formed along the direction of vapor flux with slight variations in their lengths. The SEM images of AgNRs grown on magnetic substrates at a 120 K substrate temperature indicate the zone A type of morphology. A comparison in the XRD pattern between the crystalline phase of AgNRs at room temperature and at low temperature is shown in Figure 3c (JCPDS no. 04-0783). AgNR arrays exhibit a polycrystalline structure, as indicated by the XRD patterns. As a result of the differences in peak intensities and FWHM values between the two structures, a small difference in crystallinity can be observed.

### 3.2. Flexible Substrates

Compared to conventional hard and rigid substrates, flexible substrates and chips have several advantages. They are able to conform to the underlying object, allow efficient extraction of the target molecule from complex surfaces, have highly physicochemical properties, and can be integrated into wearable devices that provide bio-detection and health-monitoring services. Polydimethylsiloxane (PDMS) and polyethylene terephthalate (PET) sheets are the most common substrates used for the growth of AgNR on flexible substrates [76,77,78]. Singh et al. prepared a mechanical-strain-resistant AgNR on flexible PDMS and PET substrates [76]. The SERS response of these flexible SERS substrates was comparable to the AgNR on conventional glass slides. Flexible SERS substrates are capable of withstanding high tensile strain (ε) (30%) without losing their SERS performance. However, similar bending can reduce SERS performance by up to 13%. Kumar et al. fabricated flexible and robust SERS-active substrates by embedding the AgNR into the PDMS. Peipei and Young presented a flexible nanoprobe for high-performance real-time SPR biosensing. They constructed plasmonic optical fiber by transferring the metallic nanostructure from patterned templates to optical fiber using epoxy and reported a high figure of merit for SPR sensors.

### 3.3. Multilayer Substrates

**Figure 4:**
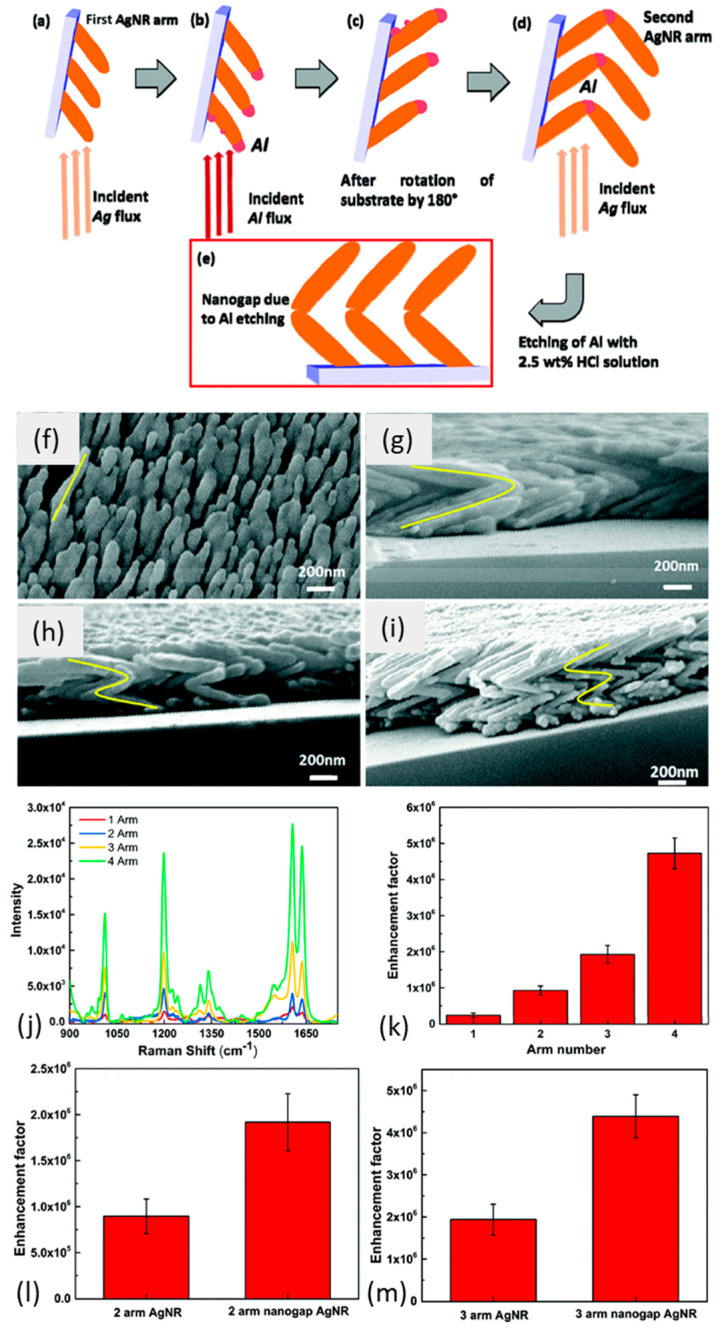
(**a**–**e**) An illustration of the fabrication of nano-porous zig-zag AgNR arrays; SEM images of zig-zag AgNR arrays on silicon substrate with: (**f**) 1 arm; (**g**) 2 arms; (**h**) 3 arms; and (**i**) 4 arms, respectively. Yellow lines show the approximate arm positions. (**j**) BPE SERS spectra (0.1 mM) acquired over zig-zag AgNR arrays with different numbers of arms. (**k**) Enhancement factor for the BPE SERS peak at 1200 cm^−1^ as a function of arm number. Calculated enhancement factor values for (**l**) 2-arm zig-zag AgNRs and 3-arm nanogap AgNR arrays and (**m**) 3-arm zig-zag AgNRs and 3-arm nanogap AgNR arrays. Reproduced with permission from [64].

Increased sensitivity and strong field enhancement can be achieved in metal–dielectric–metal multilayer structures [79]. Suzuki et al. fabricated Ag and Au nanorods with quasi-parallel principal axes on a SiO_2_ shape control layer (SCL) with a strongly anisotropic surface morphology [80,81]. They also demonstrated that the morphological and optical properties could be tuned by the deposition conditions without any pre- or post-treatment. Additionally, these structures supporting surface waves such as Bloch surface waves (BSW) could be used for advanced biosensing applications with enhanced sensitivity for protein interaction detection [82]. Multilayered structures have been used not only to tune the plasmon resonance but also to increase the number of hotspots [83,84]. Rajput et al. fabricated four-arm zig-zag Ag nanostructures of Ag and Al, with Al deposited on the edges of the zig-zag structure during growth [64]. The researchers etched less stable Al using a 2.5 wt% HCl solution from the Ag–Al zig-zag structure to generate intra-particle gaps, as depicted in Figure 4. These gaps at the elbows of the nanorods acted as additional hotspots while maintaining the zig-zag morphology. In their study, the SERS performance increased with the number of the increasing arms, which was attributed to the increased hot spots along the edges of the elbows of the zig-zag structure. The SERS performance further improved in the generation of nanoporous zig-zag Ag nanostructures obtained via de-alloying, and a high enhancement factor of ∼10^6^ was observed. These Ag structures are further under study for their applications in enhanced biosensing and early disease detection. Sun et al. constructed a sensitive biosensor based on self-assembled multilayer film for organophosphate pesticide using CdTe as a fluorescence probe. This multilayer film showed 100 times the sensitivity of CdTe in aqueous solution. Bhalla et al. created a new fabrication approach by exploiting the reactive plasma environment of SF_6_ to assemble gold nanoplasmonic structures [85]. They developed mushroom-like gold-topped nanostructures on SiO_2_ substrate. They used it as an LSPR chip for generic bioassay characterization and applications. A uniform protein coating was applied over the LSPR chip, achieving an LOD of 66 zM for biomolecules. These recent multilayered fabricated chips and nanostructures, therefore, are reported to show good stability and easy regeneration, along with increased bio-sensitivity. These can be employed as a promising platform for point-of-care diagnostics.

### 3.4. Patterned Substrates

**Figure 5:**
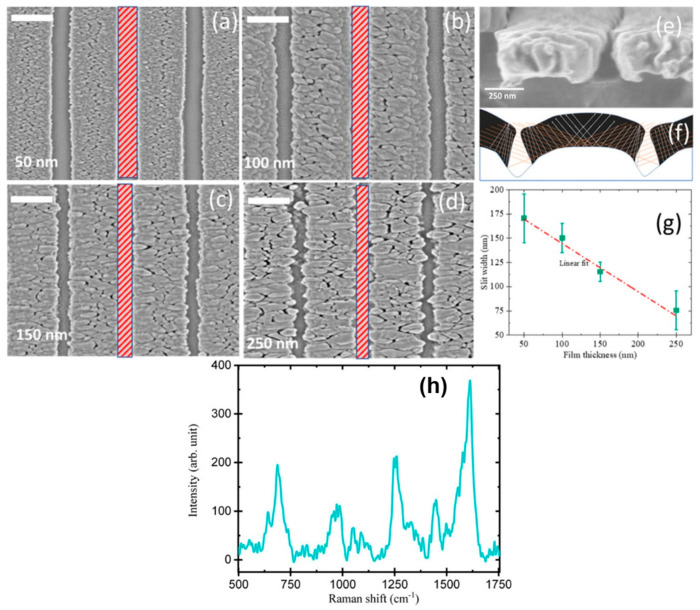
SEM images of Ag nanoslit substrates (top view) with thicknesses of (**a**) 50 nm, (**b**) 100 nm, (**c**) 150 nm, and (**d**) 250 nm. (**e**) Cross-sectional view of 100 nm thickness. (**f**) Net thickness of the film deposited at different substrate points (black region) with orange dotted lines showing incident flux direction and gray arrows showing columnar growth direction. (**g**) Plot of slit width vs. film thickness with a scale bar of 250 nm. (**h**) SERS spectra of 0.06 mg/mL bilirubin over Ag nanoslit substrate corresponding to 100 nm thickness. Reproduced with permission from [87].

Patterned substrates have been demonstrated by various groups; periodically arranged nanostructures might result in enhancement in the localized electric field of plasmonic metals compared to that of randomly arranged nanostructures employed for optical sensing applications [86]. Studies have been performed to couple the periodicity of the grating with the metal nanostructure [87], localize the deposition within a micro-well, [88,89] formation of a hexagonal lattice of nanorods, [90,91] and couple discrete metal nanoparticles to photonic-crystal surface-resonant modes [92]. Researchers have demonstrated the feasibility of patterned substrate formation by combining GLAD and electron beam lithography; thus, they have designed numerous patterned structures in which structure period and structure diameter can be adjusted in a controlled manner, hence controlling the sensitivity [86]. Therefore, this type of substrate can be useful for numerous biosensing applications where patterned structure dimensions play an important role. Anisotropic organometallic halide perovskite nanowalls fabricated using the GLAD technique have recently been reported [93]. Their high plasmonic biosensing ability can be extracted and employed for early and low-cost efficient detection and analysis. Lee et al. [94] developed a unique 3-dimensional patterned substrate employed in ultra-thin-layer chromatography (UTLC) and SERS-based sensing consisting of Si nanowire arrays fabricated using a facile photolithographic and chemical etching technique, and further decorated it with Ag nano-dendrites. These patterned substrates can be further employed for SERS-based study for the distinction and detection of diseases and their co-infections. These multifunctional substrates could simultaneously be used for target localization, mixture separation and label free detection, and therefore, could be readily employed for biochemical assays and food safety. Kumar et al. proposed a flexible nanoslit-based sensor using patterns on periodic optical disks employing imprint lithography for periodic pattern printing on polydimethylsiloxane (PDMS) [95]. Patterned PDMS was obtained using a DVD-R disk and Ag was deposited over the patterned substrates using dynamic oblique-angle deposition at an angle 76° The sample was rotated by 180o after 5 nm deposition to form a nanoslit in the grating valley as a result of the shadowing effect. Films of thickness 50–400 nm were finally obtained. Figure 5 shows the SEM images (top view) of Ag nanoslit samples of varying thickness. They further investigated the SERS-based biosensing capability of these substrates using a bilirubin solution. Therefore, patterned substrates can be used as highly sensitive multipurpose substrates in biochemical sensing.

### 3.5. Coated and Co-Deposited Nanostructured Thin-Film Substrates

The practical application of Ag nanostructures has been limited by their chemical instability due to the oxidation, sulfuration, and etching of Ag. Alloyed or coated substrates made via the co-deposition of two or more materials have been studied for increased performance and shelf life [95]. There are various reports on increasing shelf life without compromising the sensing performance of a substrate either by coating the Ag with an ultra-thin layer of graphene, SiO_2_, [96] Al_2_O_3_, [97] or by fabricating bi-metallic nanostructures [95,98,99,100]. The use of low-temperature atomic layer deposition (ALD) to wrap AgNR arrays with a very thin but dense Al_2_O_3_ layer can improve their robustness in morphology up to 400 °C, and can provide surface coverage that stabilizes SERS activity in air without reducing their sensitivity to SERS.

### 3.6. Recyclable Chip and Substrates

Most of the chips and substrates fabricated for sensing applications are for one-time use only, and given the value of noble metals, these cannot be fully explored as a routine analytical technique. Much study has, therefore, been focused on developing reusable substrates [101]. Kumar et al. fabricated a reproducible and reusable Ag nanoparticle-decorated TiO_2_ SERS substrate since TiO_2_ is renowned for its photocatalytic properties. The intensity of the Raman signal decreases rapidly in the presence of UV light because the dye concentration decreases rapidly. Approximately 150 min after illumination with UV light, these Ag-TiO_2_ nanorod SERS substrates recover completely. Hu et al. [102] reported a highly recyclable and sensitive biosensing platform comprising carbon-doped TiO_2_ nanotube arrays. These can be refreshed and regenerated photo-catalytically, maintaining high selectivity and sensitivity when irradiated with UV or infrared light. Hunt and Armani [103] developed a new idea to generate recyclable optical microcavity via a dry chemistry method using oxygen plasma treatment. This recycling treatment, therefore, helped in reducing cost and preventing harsh and wet chemistry treatment for recycling.

### 3.7. Microfluidics-Based Substrates

There is tremendous interest in developing nanofluidic channels with a large surface-to-volume ratio that exhibit localized surface plasmon resonance due to the strong interaction between the fluids and the channel walls [104]. The advantages of the nanofluidic and microfluidic platforms are: reduced unwanted and nonspecific binding, and detection time minimization by studying multiple analytes or ligand interactions using a single device [105]. By using these nanofluidic channels with plasmonic nanostructures, SERS signals from molecules trapped near electromagnetic hotspots were enhanced. A monolayer of Ag film over silica nanospheres (AgFONs) was fabricated on a glass surface and covered with a polydimethylsiloxane (PDMS) slab. This amplifies SERS by a factor of ten or more, allowing label-free detection of important neurotransmitters in the nanomolar range, rather than using only plasmon resonance.

## 4. Biosensing Applications of GLAD-Fabricated Nanostructures

### 4.1. SPR-Based Detection

Surface plasmon resonance is a powerful analytical method for detecting the high sensitivity of biomolecules, and is based on plasmonic materials’ optical properties. The SPR method has shown great promise with large sensitivity for biomolecular applications [106], especially for real-time biomolecule interaction, protein action, antigen, and nucleic acid detection [107,108,109]. SPR-based sensors are useful for label-free detection because of the very high sensitivity of the surface plasmon polaritons (SPP) to changes in the local environment in the measurement of both refractive indices and dielectric constants [110,111]. SPR-based biosensors possess the advantage of high versatility and can be tailored for the detection of a vast number of analytes since this type of detection does not demand any special characteristic of the bioanalyte, such as fluorescence properties, or absorption or scattering bands. Additionally, SPR-based bio-detection does not require analytes or the biomolecules to possess fluorescent or radioactive labels.

**Figure 6:**
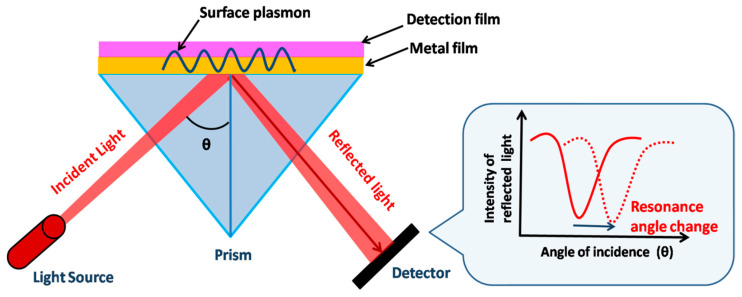
Schematic of SPR-based detection of biomolecules using Kretschmann configuration. The dip in the resonance peak shifts due to the change in the refractive index.

The primary sensing phenomenon in SPR is related to propagating surface plasmon polaritons. A surface plasmon polariton (SPP) is an electromagnetic wave propagating at the metal–dielectric boundary. The electromagnetic field of an SPP confined at the metal–dielectric interface decreases exponentially in both metals and dielectrics [112]. The dielectric constant of the propagating surface plasmon wave is highly sensitive to changes in the local refractive index of the dielectric. This property is the underlying principle of SPR biosensors. Metal-binding molecules or the recognition element attached to the surface of the metal capture the analyte, resulting in a change in the local refractive index at the metal surface. This change in the local refractive index creates a change in the propagation constant, which can be easily measured using various methods112. Figure 6 shows the underlying mechanism followed during SPR sensing using the Kretschmann configuration.

Recently, new strategies, such as the generation of frictional charges using triboelectric surfaces, have been employed for the detection and generation of localized surface plasmon resonance (LSPR) wavelength shifts in large-area Au nanostructured surfaces [113]. These LSPs with frictional charges, using recent methods such as spectroscopic and triboelectric measurements, have been found to show a complex interplay of bioconjugation and bio-complex detection. These budding strategies in combination with GLAD-fabricated nanostructures can be employed for enhanced biosensing detection and applications. GLAD-fabricated metallic thin films facilitate the development of SPR- and LSPR-based biosensors, possessing the advantage of a considerable increment in sensitivity. These nano-sculptured thin films have been demonstrated, both theoretically and experimentally, to possess high SPR sensitivity [114,115]. Additionally, their periodicity, gap, etc., can be tailored in a desired way, hence the binding of bioanalytes and sensitivity enhancement [115].

#### 4.1.1. Bi-Material-Based SPR Enhancement

The properties of two different materials favorable for experimental sensing studies can be exploited together through the fabrication of biomaterial GLAD nanostructures. Additionally, using biomaterials also helps to eliminate the unfavorable properties of either of the two materials; for example, silver is highly plasmonic and provides a sharp SPR curve with high sensitivity but becomes easily oxidized. The oxidation of silver can be eliminated if a thin layer of some inert material is used over its surface. Graphene, possessing high impermeability [116] for gases and easy adsorption [117] ability for biomolecules, can be used to coat Silver to provide high SPR sensitivity. Wu et al. reported an SPR-based graphene biosensor in which the graphene layer was coated over a gold sheet. The sensitivity of the chip was shown to increase several times through calculations compared to the conventional gold film used for SPR biosensing. In another work, Choi et al. [118] presented a numerical model showing a layer of graphene coated over a 60 nm silver film. The SPR sensitivity was shown to increase several times due to the graphene coating and also to prevent the oxidation of silver. Over the graphene surface, a bimolecular DNA hybridization was modeled as a thin dielectric layer. The group showed improvement in the SPR bimolecular sensitivity of the graphene-coated substrate compared to conventional gold film chips employed for SPR biosensing applications. Graphene adds to the SPR sensitivity in comparison to the gold film due to its better adsorbing properties of biomolecules; this is due to the pi interactions between the biomolecules’ carbon ring structure and graphene hexagonal cells [119,120]. Gold/zinc oxide (Au/ZnO), as well as gold/chromium (Au/Cr)-based nanocomposite thin films, have also been studied and were used effectively for enhancing SPR performance for the detection of a carbohydrate antigen, which is a tumor marker for breast cancer. The linear range for the SPR sensor was shown to increase from 1 to 40 U/mL [120]. Therefore, bimetal GLAD structures can be used to increase SPR-based bio-sensitivity along with increasing the inertness of the metallic structures.

#### 4.1.2. Nanocolumnar Thin-Film-Based SPR Chips

**Figure 7:**
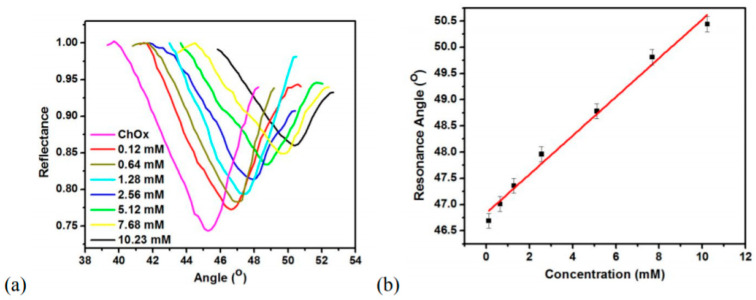
(**a**) SPR reflectance curve for ChOx/ZnO/Au prism corresponding to different cholesterol concentrations. (**b**) Calibration curve for variation in resonance angle with cholesterol concentrations. Reproduced with permission from [122].

As discussed in the earlier section, thin-film nanocolumns fabricated using the GLAD technique provide high sensitivity for the detection of bioanalytes. They are easy to fabricate, possess a high surface –volume ratio, require less material, and are broadly used as SPR chips. The use of these metallic nanocolumnar thin-film biosensors enhances both angular as well as spectral sensitivity due to increased roughness and porosity, and induces SPR broadening due to scattering losses inside the porous metallic film [120,121]. Kaur et al. [122] reported ZnO thin-film-based SPR detection for cholesterol biosensing applications. ZnO was coated over the gold film using the GLAD technique. ZnO/Au prism was used in the Kretschmann configuration. The cholesterol oxidase (ChOx) enzyme had been immobilized over the ZnO surface. PDMS microchannels fabricated over the ChOx/ZnO/Au/prism setup were used to pass different cholesterol concentrations, and SPR reflectance was obtained for both static and running mode, showing high SPR sensitivity. Figure 7a shows the SPR reflectance curves for various cholesterol concentrations over the ChOx/ZnO/Au prism, and Figure 7b shows the calibration curve for the variation in the resonance angle with cholesterol concentrations. However, in most of the work discussed above employing SPR detection using thin metallic films, the sensor chips were fabricated as planar thin films using GLAD-based techniques. The experimental use and applications of GLAD-based columnar thin films using different structural morphologies are still limited for biosensing applications. The effect of porosity on the SPR dip for different metallic nano-columnar thin films (Al, Ag, and Au) fabricated via GLAD using Kretschmann configuration has also been studied. The SPR dip widens with an increase in the porosity of the thin film, and finally, disappears near the TIR region. This group showed the vast possibility of SPR-based biosensors utilizing the porosity of the metals with increased sensitivity compared to nonporous films.

**Figure 8:**
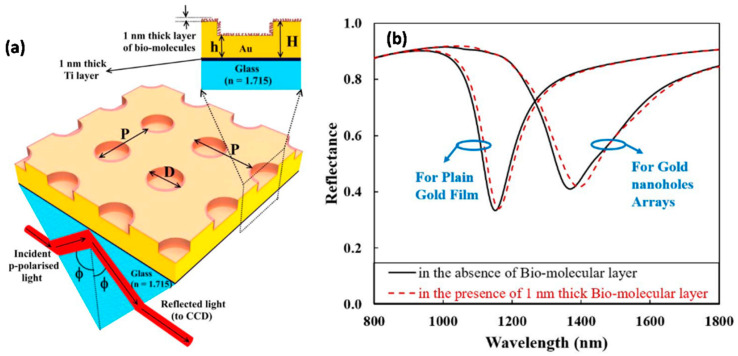
(**a**) Schematic diagram of circular nanohole array over the gold film. P, D, h, and H represent array period, array diameter, gold film thickness below the nanohole, and gold film thickness without the nanohole, respectively. This array is further coated with a 1 nm thick layer of biomolecules. (**b**) Reflectance spectra calculated for a planar gold film of thickness 60 nm and for gold film structure array with a period (P) and diameter (D) of nanohole array of 50 nm and 30 nm, respectively; gold film thickness without nanohole (H) of 60 nm; and gold film thickness below nanoholes of 40 nm. Reproduced with permission from [56].

Shalabney et al. [123] have explained this phenomenon very well both via theoretical calculations as well as experimentally. They carried out SPR measurements in Kretschmann configuration for GLAD-fabricated Ag nano-columnar thin films for different values of β and studied the SPR sensitivity in both angular as well as spectral modes. They indicated that for more porous films, the resonance wavelength shows a clear redshift of about 15 nm with respect to less porous films, and hence, higher sensitivity. Further, a theoretical calculation of the dielectric tensor of the material was carried out. There was an enhancement in the electromagnetic field intensity distribution as there was an increase in the volume interaction of the evanescent field in both the columnar void region and the sample area. These nanocolumnar thin films provide a high increment in sensitivity compared to planar metallic films. Agrawal et al. [114] presented a 2D nano-pillar array with nano-gaps for biosensing. They used rigorous coupled-wave analysis (RCWA) simulations to study the SPR sensitivity. The biosensor performance was studied in terms of sensitivity, characterized by spectral shift Δλ. The effect of polarization of the light incident was studied using simulations, which showed that nano-pillars’ biosensing capability is independent of incoming light. The sensitivity of SPR chips can be most suitably defined in terms of differential reflectance amplitude (DRA) since the sensor performance also depends on reflectance contrast along with reflectance change. Agrawal et al. [56] also demonstrated, in another work, the increased sensitivity of the SPR detection of nanohole arrays of gold compared to planar gold film (Figure 8). The SPR sensitivity of the biosensor had been calculated in terms of DRA. They carried out RCWA simulations to study the effect of variation in the diameter and period of the nanoholes. Byun et al. [124] experimentally confirmed the high SPR bio-sensitivity of a gold nanowire array compared to conventional gold film. They used an angular interrogation scheme to perform biosensor characterization and showed that their results were in agreement with RCWA simulated results. They fabricated gold nanowires with two different periods—200 nm and 500 nm—with each showing increased sensitivity of 44% and 31%, respectively, when evaluated using ethanol of varying concentrations.

Combining the two different ideas discussed above, one may suggest the fabrication of nanocolumnar thin films consisting of GLAD-engineered structures of different morphologies combined with other materials (ZnO, graphene, and gold) to increase bio-sensitivity. This may lead to us to ultra-high sophisticated SPR biosensors with multiple-fold sensitivity increments.

#### 4.1.3. Microfluidics-Based SPR Chips

Microfluidic microchannels and systems are mainly employed for the SPR imaging approach. The advantages of the microfluidic platform are: reduced nonspecific binding and detection time minimization by studying multiple analytes or ligand interactions using a single device [105]. SPR-based microfluidic assays have been developed for various biomolecular interactions such as antibody-binding targets [125], multi ligand/analyte biosensing [126], and foodborne pathogen detection [127]. Malic et.al. [128] reported a multichannel droplet-based SPR detection platform comprising electrowetting on a dielectric microfluidic device for SPR imaging. The device was fabricated using Cr and Au layers as SPR supporters followed by the patterning of 24 um photoresist as a lift-off mask. A 50 nm Au film was etched to define a thin Au layer as ground. So, a spot-by-spot detection achievement was employed. Natarajan et al. [129] reported a 3D microfluidic system for protein sample deposition to discrete spots of a target. They showed exposure of each spot to a larger sample volume due to continuous flow and increased uniformity of the spots, and hence, expanded the protein array applications for protein printing. A compact and robust microfluidic flow cell for SiO_2_ film followed by a PDMS microfluidic surface has been developed [130]. This biosensor flow has the advantage of operation up to 185 kPa in an aqueous environment. A unique droplet-based SPR biosensing platform coupled to a surface acoustic wave microfluidic system (SAW) has also been reported [131]. Surface acoustic wave excitation in continuous mode for different levels of power generation was studied. Droplet-based microfluidic SPR systems possess the advantage of limiting mass transport in comparison to conventional microfluidic flow.

Though most microfluidic systems focus on on-chip fabrication for SPR imaging, the major challenges of cost-effectiveness and the simplicity and compatibility of the detection and monitoring of SPR signals still require further effort and research. GLAD-fabricated arrays and chips can be very promising candidates for highly sensitive SPR imaging purposes.

### 4.2. SERS-Based Biosensing

Surface-enhanced Raman scattering (SERS) is the enormous amplification of Raman signals from molecules, by several orders of magnitude, when adsorbed on metal colloidal nanoparticles or a rough metal surface, discovered in 1974 by Fleischmann et al. [132,133]. The detection of analyte molecules even at a single-molecule level can be realized by employing the SERS technique, which expands its practical applications [134]. SERS has found applications in various fields, including the detection of trace chemicals [135] such as dye molecules, food additives, [136] and pesticides, [77] and in bioanalysis, medical diagnosis, [36] the detection of biomolecules, [137] cancer diagnosis, [138] in vivo molecular probing in live cells, [139] and explosives detection [140]. SERS substrates are the nanostructured platform that supports plasmon resonance and amplifies Raman signals [141,142,143] and are broadly classified as random morphology or ordered/periodic SERS substrates. Random morphology SERS substrates (which include roughened electrodes, metallic silver and gold colloids, and metal-island film on a planar substrate) are inhomogeneous and are not highly reproducible [144]. Periodic arrays of metallic nanostructures (using nanolithography and other physical vapor deposition techniques) can overcome this issue by providing uniform and controlled morphology of SERS substrates [145].

SERS has become a powerful technique for chemical and biological sensing applications due to its excellent sensitivity and specificity. Uniform and highly reproducible SERS substrates with batch-to-batch reproducibility that have a SERS signal variation of less than 15% can be fabricated using the GLAD technique [146]. Using SERS multivariate statistical analysis, various pathogen species or even strains can be differentiated. Therefore, extrinsic detection using Raman labels is more widely explored.

#### 4.2.1. Detection of Pathogens

**Figure 9:**
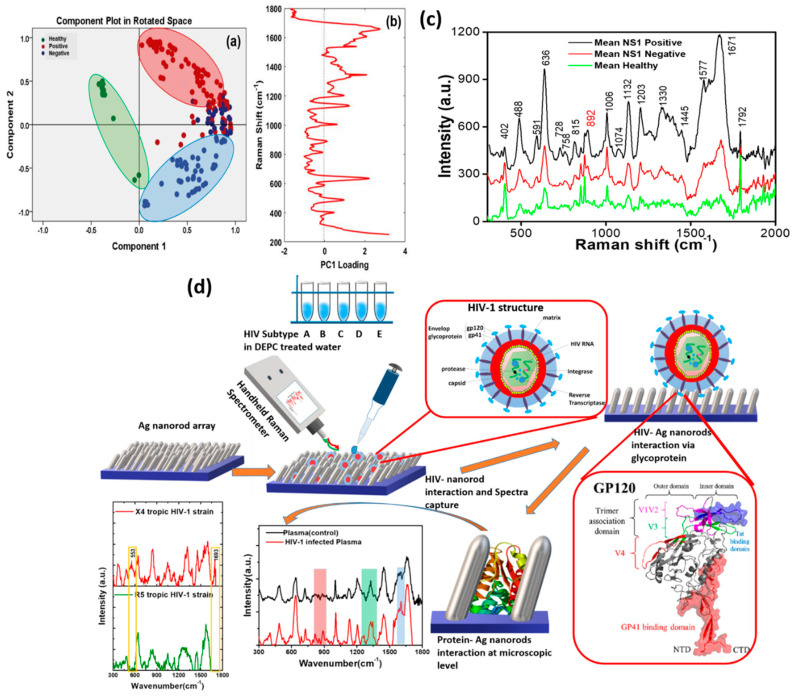
(**a**) Figure showing principal component 1 versus principal component 2 for three different groups of samples (antigen NS1-positive, -negative, and healthy). (**b**) Graph showing Raman shifts with the PC1 loading of plot (**a**). (**c**) SERS spectra obtained for the healthy, NS1-negative, and NS1-positive sera collected from the patients (each spectrum is an average of 20 spectra). Reproduced with permission from [36]. (**d**) Schematic diagram showing rapid handheld SERS platform for early HIV detection. Reproduced with permission from [54].

SERS substrates fabricated via GLAD have been utilized for the detection of human pathogens, particularly respiratory syncytial virus, human immunodeficiency virus, and rotavirus; the bacteria of mycoplasma pneumonia, tuberculosis, E. coli, and Pseudomonas aeruginosa; and the identification and classification of various virus strains. [54] Gahlaut et al. demonstrated the diagnosis of dengue (DENV) from clinical serum samples using a Ag nanorod array-based hand-held SERS platform [36]. Using the GLAD-deposited SERS substrate and principle component analysis (PCA), the DENV-positive, -negative, and healthy samples were analyzed and differentiated (Figure 9). They identified the SERS spectra of pure NS1 protein, both separately and in serum, and the corresponding characteristic peaks of NS1 protein. This portable, rapid, and cost-effective method can diagnose dengue early in the field, and with a high degree of sensitivity and reproducibility. Kumar et al. demonstrated a facile and straightforward method to increase the SERS enhancement of bacteria [78]. They deposited AgNRs on 30% pre-stretched PDMS using the GLAD technique and directly pipetted bacterial suspension (3 μL, 10^8^ cells mL^−1^) onto the AgNRs grown on the stretched PDMS substrate. They performed SERS measurements before and after releasing the strain of the stretched AgNR-PDMS SERS substrate. The release of the strain led to the formation of high-density hotspots in the AgNRs, allowing the bacteria and AgNRs to interact more effectively. Compared to a pre-stretched AgNR-PDMS film, these buckled AgNR-PDMS array substrates showed an approximately eleven-fold enhancement in SERS. Yadav et al. demonstrated a rapid SERS platform for rapid HIV-1 detection. The group performed a detailed study over five different HIV-1 subtypes. HIV-1 envelopes possessing glycoproteins bind to the AgNRs SERS substrate without any external reagent, thus providing specific signature peaks of HIV-1. Additionally, they showed a distinction in SERS spectra based on tropism. Their proposed technique could successfully distinguish between X-4 and R-5 tropic HIV viruses [54] (Figure 9d).

#### 4.2.2. Detection of Biomarkers

**Figure 10:**
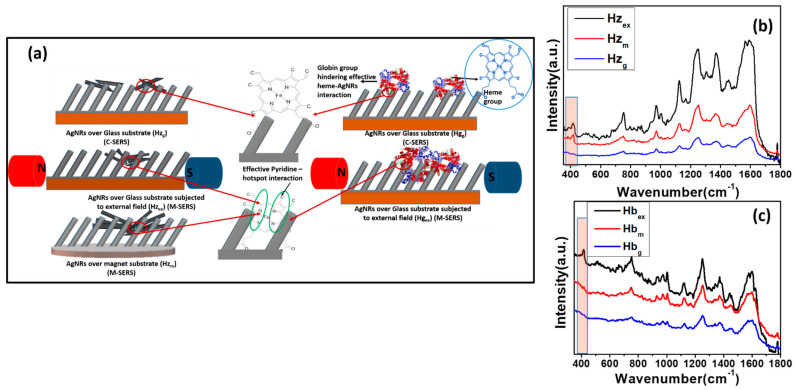
(**a**) Schematic diagram showing pyridine–AgNR interactions for the unmagnetized Hz and Hg over AgNS substrate (c-SERS), in the presence of a magnetic field (M-SERS). (**b**) Comparison of SERS spectra for Hz on AgNR arrays fabricated over glass (Hz_g_) (neodymium magnet (Hz_m_), Hz_g_ substrate kept at 0.3 T external magnetic field for 1 h (Hz_ex_)) and (**c**) Hg on AgNR arrays fabricated on glass (Hb_g_) (neodymium magnet (Hb_m_), Hb_g_ substrate kept at 0.3 T external magnetic field for 1 h (Hb_ex_)). Reproduced with permission from [63].

A biomarker is any indicator of the severity or presence of a particular disease in an organism. Kumar et al. demonstrated the potential application of Ag nanoslit (AgNS) SERS substrates through bilirubin detection. Bilirubin is a toxic metabolite of heme reduction, and excessive levels of bilirubin result in well-known symptoms of liver jaundice. The group detected the SERS signal of bilirubin with a concentration much below the normal concentration in human blood using this AgNS substrate [87]. Yadav et al. used hemozoin and exploited its magnetic properties for the early and easy detection of malaria. They presented a unique magnetic field-based surface-enhanced Raman spectroscopy (M-SERS) technique for magnetic biomarkers. SERS-active Ag nanorods were grown over magnetic neodymium substrates (0.3 T) at low temperature using the GLAD technique. Figure 10 shows the complete mechanism of M-SERS and the obtained SERS spectra. The obtained limit of detection of hemozoin for M-SERS was as low as 10^−11^ M (< 10 parasites/µL), meaning it can be employed for early-stage malaria detection [63]. Srivastava et al. developed a biosensor chip for the detection of a protein biomarker of endocrine-disrupting compounds, vitellogenin (Vg), in an aquatic environment [147]. The researchers immobilized the anti-Vg antibody on 4-Aminothiophenol-coated Ag thin films for the specific detection of Vg. They also performed a control experiment to demonstrate the specificity of the sensor towards Vg using fetuin for the control experiment, which is a glycoprotein like Vg. The SERS substrate also exhibited high stability over a period of one year. SERS-based sensors for the detection of specific biomarkers is, therefore, a well-established sensing phenomenon and can be employed with advanced substrate growth techniques for enhancing sensitivity to the single-molecule level.

#### 4.2.3. Detection of microRNA and Neurotransmitters

MicroRNAs (miRNAs) are a class of non-coding RNAs that play essential roles in regulating gene expression and also act as biomarkers for disease [148]. The evaluation of miRNA expression patterns is limited to cumbersome assays that often lack sensitivity and specificity to distinguish between different miRNA families and members. A SERS platform for the detection and classification of miRNA using GLAD was demonstrated by Driskell, and Tripp [149,150]. They detected and differentiated five unrelated miRNAs and eight members of the hsa-let-7 family using SERS. In their study, the SERS platform overcame the limitations of data normalization, empirical probe selection, and RNA labeling or amplification depending on the intrinsic stability and specificity of the reagents used in the PCR-based assays. By using SERS, microRNAs can be rapidly and specifically detected without amplification, providing a molecular fingerprint of each analyte and requiring only limited amounts of sample for analysis. Additionally, it improves comprehension of the spectrum properties of miRNAs, making it easier to interpret the results.

Oh et al. demonstrated the biosensing capability of microfluidic-based SERS substrates fabricated using GLAD by sensing two major neurotransmitters, named dopamine and gamma aminobutyric acids (GABA) [104]. Interstitial nanogaps between AgFONs and the PDMS channels were shown. Capillary force was used to inject a very small amount of analyte at a very low concentration into these nanofluidic channels. Due to the coexistence of nanofluidic stagnation points and hotspots with plasmon resonance, the SERS signals were greatly enhanced. Dopamine and GABA solutions at different concentrations were prepared in distilled water. SERS signals were detected at different concentrations in the nanofluidic channels and compared with those in the reservoir. It was found that the SERS signals increased with increasing concentrations of the neurotransmitter. While the peak intensity of GABA at 1370 cm^−1^ was 20 times higher in the nanofluidic channel than in the reservoir, the SERS peak intensity of dopamine at 1605 cm^−1^ was eight times higher in the nanofluidic channel.

#### 4.2.4. Detection of Pesticides and Chemicals

Pesticide and trace pollutant detection are essential because of the hazard to the environment and public health caused by the accretion of these harmful chemicals. SERS substrates fabricated using the GLAD technique have been extensively utilized for the detection of these harmful chemicals [76].

**Figure 11:**
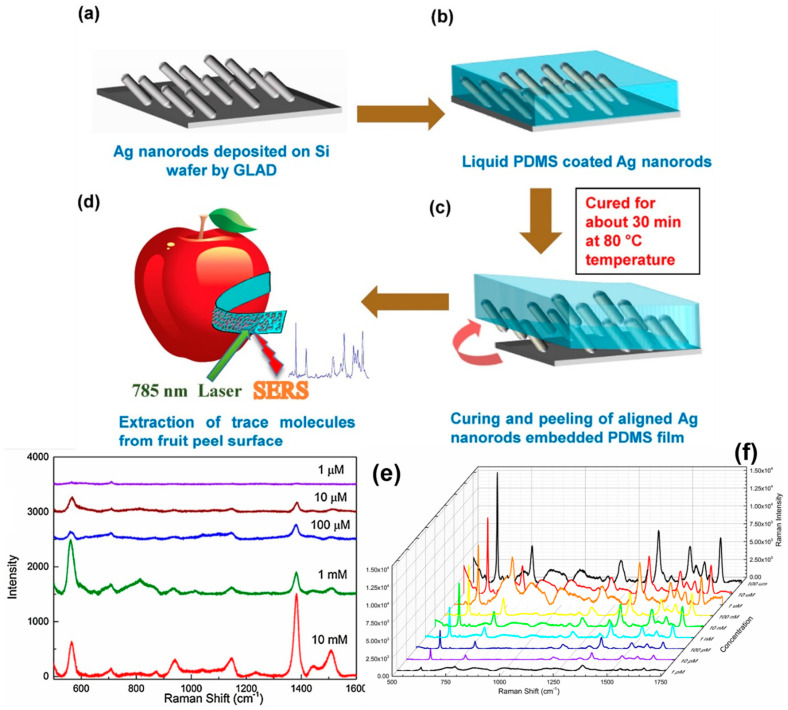
Schematic showing fabrication of AgNR-embedded PDMS SERS substrates (**a**–**d**). (**e**) SERS spectra of *thiram* pesticide. (**f**) SERS spectra of rhodamine 6G with different concentrations on AgNR-embedded substrate. Reproduced with permission from [77].

Kumar et al. demonstrated a highly optimized SERS-active substrate for the easy and rapid detection and extraction of target molecules from complex surfaces [77]. The researchers directly collected traces of the pesticides thiram and chlorpyrifos from an apple surface using AgNRs embedded in PDMS substrates using a simple “paste and peel off” method (Figure 11). They were able to detect a Raman band at 1386 cm^−1^ even at a concentration of 10^−6^ M R6G, outperforming more established methods such as high-performance liquid chromatography (HPLC). They reported a limit of detection (LOD) of thiram of 2.4 × 10^−9^ g/cm^2^, demonstrating the practical application of SERS substrates for the rapid trace detection of thiram, with an LOD much lower than the permissible limit for apple peels (~2 × 10^−6^ g/cm^2^). Hou et al. developed SiO_2_ nanorod (NR)@Au nanoparticles (NPs) for the detection of monochlorobiphenyl (CB) as low as 1 × 10^−6^ M with an enhancement factor of 10^8^ [151]. Moreover, the authors also demonstrated the reusability of the SiO_2_@Au SERS substrate in order to detect trace CB.

#### 4.2.5. Detection of Drugs and Food Adulterants

The GLAD technique for SERS substrate fabrication has been used for the trace detection of methamphetamine and its primary metabolite, amphetamine, in human urine [152,153]. The rapid onsite screening of food contamination in quality control and market surveillance applications has also been reported [154]. Nuntawong et al. proposed an alternative method for the pre-treatment of urine-based samples to prevent urea from binding to the surface of SERS-active substrates [152]. Assuming that the acidic dilatation treatments would convert the urea to the much less soluble urea nitrate, allowing precipitation from the mixture, and that the remaining urea would be negatively inhibited from adhering to the SERS-active surface, they added dilute nitric acid to the urine samples. Consequently, they performed the SERS measurements from a series of mixtures of the urine-based samples and the diluted nitric acid via a compact SERS detection system using a portable Raman spectrometer.

Sodium saccharin (SS) is a commonly used synthetic sweetener in carbonated soft drinks and beverages. Han et al. developed a sensitive SERS method for the quantification of SS in soft drinks [155]. SERS peak intensity and SS concentration in the concentration range of 0.5–100 mg/L showed an excellent linear relationship, with a LOD of 0.3 mg/L. They also performed PLS-DA analysis to determine the LODs of SS in four soft drinks—Sprite (20 mg/L), Cola (5 mg/L), Fanta (10 mg/L), and Schweppes (20 mg/L)—which were far below the national standard.

Therefore, GLAD is a versatile nanofabrication technique for the design of nanostructured thin films for SERS sensing [67]. However, there are still a number of challenges that must be overcome before SERS can be used as a common analytical technique, like HPLC, for complex food matrices and pathogens in the real world. These include the high cost of the Raman spectrometer and the lack of commercially available, low-cost SERS substrates. When SERS measures trace amounts of any analyte in a food, background interference from food components is still a major problem. Therefore, further research is needed to successfully advance the SERS approach in practical applications of food analysis and disease identification.

### 4.3. Fluorescence-Based Biosensing

Fluorescence is defined as ‘a short-lived kind of luminescence’, created as a result of electromagnetic excitation caused when light energy is absorbed in a short wavelength, and then, emits the light at a longer wavelength [156]. Fluorophores or fluorochromes are the molecule that shows fluorescence. Fluorophores emit energy in the form of light radiation or sometimes dissipate in the form of heat. The basic concept of biosensing using a fluorophore involves the coupling of target recognition with some change in the fluorescence of the reporter molecule. Different types of fluorescence biosensors can be proposed based on the nature of the sensing and binding elements [157]. The change in the intensity of fluorophores attached to the recognition element is very sensitive to the local environment and is monitored directly. This change in the fluorescence intensity may be due to a change in fluorophore–biomolecule interactions, [158] or due to fluorophore–target molecule interactions, for example, the interaction of single-stranded DNA protein binding [159]. The basic advantages of fluorescence-based biosensors are its simplicity, its ability to detect smaller ligands, the availability of many donors and acceptors, its convenient transducing optical signals, and its suitability for short distances. Metallic nanostructures are known to enhance the fluorescence of fluorophores [160,161,162]. The fluorophores interact with the plasmonic field at the metal surface, therefore increasing the local field, resulting in enhanced fluorescence intensity [160,161,163]. The metallic structures are known to increase the local electric field as well as the radiation decay rate up to a factor of 1000 [161]. The sensitivity of fluorophore detection due to combined local field increments and increased photostability is enhanced by 10^5^ [164]. Metallic nanostructures are employed for enhanced fluorescence detection of DNA [165], protein micro arrays [166], pathogens, cancer cells [167], and single species in tissue samples [168]. Enhanced fluorescence detection using chemical method-based metallic nanoparticles is, however, limited due to the inhomogeneity of the structure and non-proportion of fluorophore interaction with metals that provide enhancement. Plasmonic fluorescence enhancement by PVD-deposited metal nanostructures tends to provide increased enhancement due to the formation of periodic arrays over a large surface area. Controlled growth parameters and deposition rates may provide a large array of nano-columns with different shapes, sizes, periodicity, and interparticle separation, which can be regulated on the metal surface. Different applications of fabricated metal nanostructures in enhanced fluorescence-based bio-detection are discussed.

#### 4.3.1. Protein- and DNA-Fluorescence-Enhanced Detection

**Figure 12:**
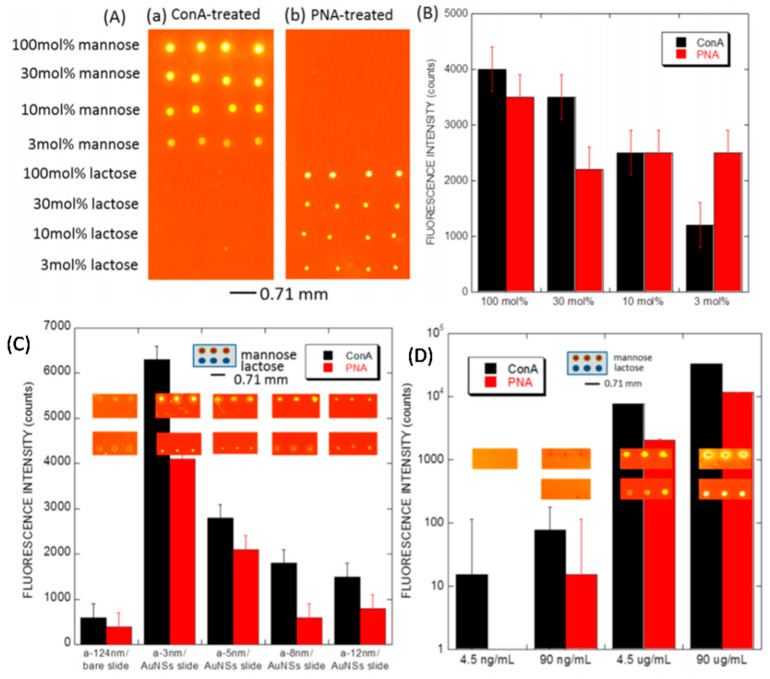
(**A**) Fluorescence images arrays spotted after interaction with (**a**) Con A(0.9 mg/mL) and (**b**) PNA (0.9 mg/mL). (**B**) Histogram of corresponding fluorescence intensity for diluted glycan spots. (**C**) Fluorescence histogram curve for spotted arrays with 100% lactose and 100% mannose after interaction with ConA and PNA (with a fluorescence exposure time of 2 s) followed by PBS rinse (**D**). Fluorescence histogram for spotted 3 nm thick Si 0.8 C0.2:H Au nanostructure array with 100% lactose and 100% mannose after ConA and PNA interaction (fluorescence exposure time of 5 s) followed by PBS rinse. Reproduced with permission from [169].

The development of optically active metallic nanostructure substrates enhancing fluorescence can be used for detection using fluorescence-tagged analytes in DNA analysis and protein detection assays. Yang et al. [169] described, in their paper, a selective and sensitive carbohydrate microarray for glycan protein interaction detection. They deposited gold nanostructures using thermal evaporation, coating it further with a silicon carbon alloy layer, which was subjected to a surface-linked azide group for immobilization of glycan through the formation of covalent bonds. Investigation of the enhanced fluorescence-sensing ability of metal nanostructures via the characterization of carbohydrate lectin interactions has been conducted. The fluorescence-labeled lectins Concanavalin A (ConA) and peanut agglutinin (PNA) were chosen for specific interaction with lactose- and mannose-modified LSPR interfaces (Figure 12). In another work, Li et al. [166] presented highly sensitive fluorescence-enhanced detection in a silver metal microarray. Detection of IgE and streptavidin has been conducted using aptamers for tag-C preparation. The silver nanostructures formed using tags and fluorescence-enhanced solutions provided excellent enhancement. Fluorescence enhancement studies for DNA microarrays, with the help of GLAD-deposited silver nanorods of various lengths, have been performed; the highest enhancement of 200 folds is reported for Ag nanorods of 500 nm in length, which is multiple times higher than the commercially available amine slide [165]. Zang et al. [170] reported that fluorescence intensity enhancement increased 114 times using a GLAD-fabricated TiO_2_ nanorod film applied over the surface of a label-free photonic crystal device. The fluorescence enhancement of the device was studied both experimentally and theoretically (RCWA simulations) for cy5-conjugated streptavidin application, and the enhancement of the signal was compared with the device without nanorods. These studies showed that GLAD-fabricated nanostructures can be an invaluable tool to study sensitive fluorescence enhancement for bio-detection and similar devices can be designed for sensing applications.

#### 4.3.2. Detection of Pathogens

**Figure 13:**
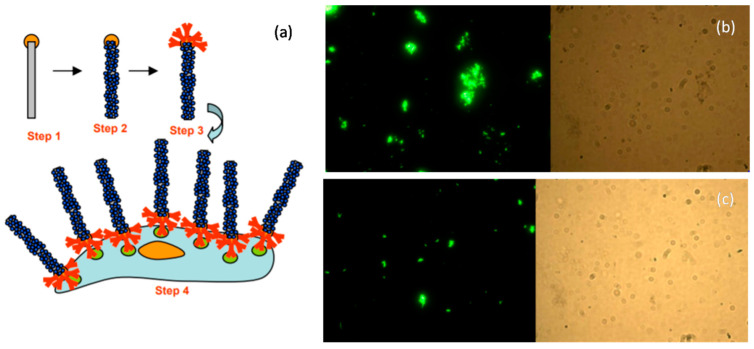
(**a**) Step-by-step process for Salmonella detection using Au/Si rods comprising nanorod fabrication, dye immobilization, antibody conjugation, and bacteria detection through antigen–antibody conjugation, respectively. Fluorescence microscopic images with BSA blocking under fluorescent light and white light for (**b**) anti-salmonella/Au/Si nanorods and fixed salmonella and (**c**) Au/Si nanorods and fixed salmonella. Reproduced with permission from [172].

The rapid, cost-effective, selective, and sensitive detection of bacterial pathogens is very important for disease treatment, preventing bioterrorism, diagnosis, and foodborne disease identification. However, the majority of methods used are laborious, costly, and time-consuming. The fluorescence-based detection of these pathogens using nanostructures, however, can overcome these limitations. Geddes et al. [171] patented a method in which they provided a system whereby metal particles placed over a quartz substrate are attached to a biomolecular probe that possesses an affinity for the target pathogen. The binding target pathogen in contact with the biomolecular probe causes fluorophores to be at a sufficient distance from the metal structure, resulting in enhanced fluorescence emission when irradiated by a source. Therefore, they developed an entire platform for the rapid and sensitive identification of target pathogens via fluorescence emission. Park et al. [172] developed a novel biosensor for rapid pathogen detection using hetero-nanorods. The GLAD-fabricated silica nanorods were gold-sputtered and the dye immobilized via bond formation between the primary amine group and the dye ester. This was followed by antibody conjugation and bacteria detection. The entire detection process is shown in steps in Figure 13. The dye molecules attached to the nanorods resulted in an enhanced fluorescence signal and Salmonella detection using microscopic imaging. Hence, GLAD-fabricated nanostructures of different materials can be employed for fluorescence-based biosensing to detect foodborne pathogenic bacteria.

#### 4.3.3. Single-Molecule and Tissue Detection

Single-molecule detection techniques are, nowadays, vastly explored. These techniques provide a new powerful idea to explore biochemical and life science components at the elementary level. However, conventional optical methods face diffraction-based limitations [173]. Fluorescence-based detection enhancing the signals, even for very small analyte concentrations, opens up new possibilities for single-molecule detection.

Zhang et al. [174] reported target molecule imaging using fluorescent material probes. They synthesized silica metal nanoshells encapsulated by Ru(by)_3_^2+^ to obtain fluorescence properties. Avidin silver complexes were formed, and imaging measurements indicated that individual complexes could be distinctly isolated while the tissue remained stained by organic dyes. Hence, the group successfully demonstrated that metal-based nanoprobes could be employed for single-target-molecule tissue detection during fluorescence imaging. Metal nanoprobes have also been used to detect CCR5 receptors over the cell surface using a covalent bond between anti CCR5 monoclonal antibodies and silver nanoparticles [168]. The mAb-metal complex showed a single nanoparticle emission. Punj et al. [175] described that plasmonic nanoparticle dimer antennas can be extremely promising for single-molecule enhanced fluorescence detection at micromolar concentrations. They quantified the fluorescence enhancement and detection volume for various self-assembled nanoantennas using fluorescence spectroscopy. Hence, the combination of metallic nanostructures along with fluorescence enhancement can be used for an extended variety of single-molecule detection applications.

### 4.4. Colorimetric- and Wettability-Based Detection

**Figure 14:**
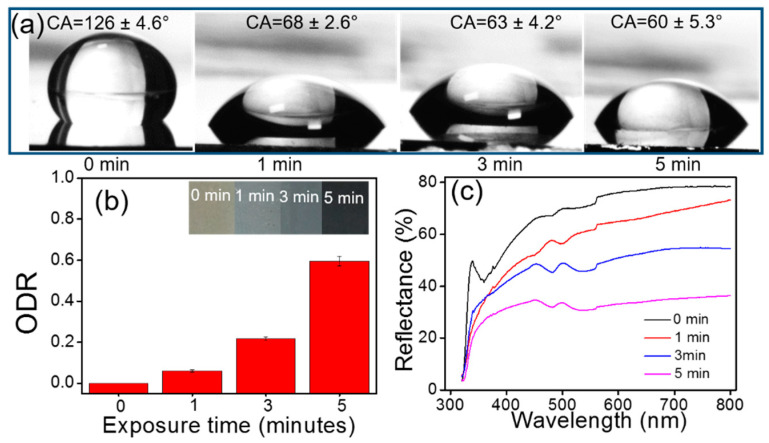
Variation in (**a**) water contact angle and (**b**) optical darkness ratio (ODR); inset shows respective photographs. (**c**) Surface reflectance of AgNR array samples after 5 ppm H_2_S exposure times of 1, 3 and 5 min. Reproduced with permission from [185].

Sensing based on the change in color and surface wettability has also been realized on GLAD substrates, especially plasmonic metallic arrays. Silver has the highest reflectance among all metals, at over 97% throughout most of the visible region and about 99% in the IR region. Pure silver looks shiny and whitish in color, as seen by the naked eye. However, silver nanoparticles exhibit a plasmonic effect with a very high extinction coefficient, and plasmonic absorption lies in the visible range and varies with particle size and shape, interparticle separation, and the refractive index of the surrounding medium [176]. A homogenous colloidal solution of nanoparticles of a particular size scatters a specific wavelength of light, which makes them promising for distinguishable colorimetric visual readout sensors. Such properties of metal nanoparticles enable them to be exploited in various analytical tools, e.g., the absorbance or fluorescence spectroscopy. Colorimetric-based assays have been developed by illustrating changes in the color associated with the aggregation of noble metal nanoparticles [177,178,179]. However, the employment of GLAD-fabricated silver nano-columnar thin film in such sensing applications is limited. The pristine aligned AgNR array fabricated by GLAD in a high vacuum looks bright due to the multiple scattering and multimode localized surface plasmon. In addition to optical-based sensing, these nanorod substrates have also been studied for gas and biosensing by exploiting their novel characteristic colorimetric properties. The chemistry between silver and sulfur has been studied by different groups [180,181,182]. Graedel et al. extensively explored the reaction between silver- and sulfur-containing gaseous molecules and found out the dependence of the reaction rate on relative humidity. Thereafter, Chen et al. demonstrated the use of AgNP films as H_2_S gas sensors. According to their findings, the reaction between AgNPs and H_2_S gas follows a first-order reaction rate law and is proportional to the 1.3 power of the H_2_S gas concentration [182,183,184]. This relationship was used to determine the H_2_S gas concentration under ambient conditions. The intensity of the LSPR peak of the AgNP’s films decreases and exhibits a shift upon exposure to H_2_S gas. Though they have several applications in art conservation, AgNPs are used to detect the emission of H_2_S from degraded materials, e.g, aged wool fabrics, rubbers, etc. under ambient conditions. A GLAD-fabricated AgNR array was employed as an H_2_S gas sensor by Gahlaut et al. [185]. The authors demonstrated a dual-mode, i.e., colorimetric- as well as wettability-based detection, using a mobile app which calculates the change in greyscale intensity of the AgNR array upon exposure to H_2_S gas. The optical darkness ratio (ODR) was defined as the relative change in the average pixel intensity of pristine and exposed AgNR arrays. The variation in ODR values is shown in Figure 14b.

**Figure 15:**
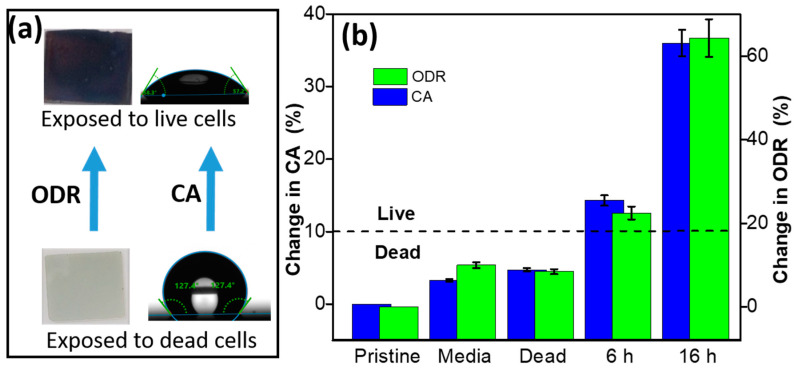
Determination of live and dead *E. coli* bacteria using a change in ODR and CA. (**a**) A digital photograph of the sensing array and water droplet on the array for the ODR and CA measurements. (**b**) Graph showing the percentage change in ODR and CA for pristine array and after 6 h and 16 h of culture. The media and heat-killed bacteria were considered as a control. A demarcation line is drawn representing the threshold values above which the live bacteria are distinguishable from dead bacteria. Reproduced with permission from [38].

The limit of detection and response time of the detector were determined as 5 ppm and 30 sec, respectively. The method was also applied to the detection of H_2_S emission from aged wool fabric placed in textile museums and art galleries to prevent artifacts made of silver. In another report [38], the same group also demonstrated the determination of viability and antibiotic resistance in bacterial cells based on the colorimetric change in an AgNR array, as shown in Figure 15. The nanorod array was found to be highly sensitive and selective toward H_2_S gas in the presence of sub-ppm concentrations under ambient conditions.

Ahn et al. [186] introduced a highly inexpensive and facile colorimetric system for selective H_2_S detection in living cells using a silver-embedded Nafion/polyvinylpyrrolidone (PVP) membrane. The membrane was applied to a polystyrene microplate cover. A known concentration of the H2_S_-donor Na_2_S solution was used to evaluate the performance of the in vitro colorimetric detection assay with the silver/Nafion/PVP membrane, which was performed both at room temperature and at 37 °C in an incubator containing 5% CO_2_. Last but not least, they used the silver/Nafion/PVP membrane to determine the endogenous H_2_S concentration in live C6 glioma cells stimulated with and without Cys and L-homocysteine. The rationale behind the detection of H_2_S gas was that silver reacts with the gas to form Ag_2_S, which has a brown color.

Hao et al. [187] developed a highly sensitive H_2_S detection platform by using single gold and silver core-shell nanosphere for mapping the local distribution of H_2_S in living cells. The concept of sensing was the same colorimetric variation of the Ag shell in the presence of sulfide. The formation of Ag_2_S on the surface led to a large difference in the refractive index between Ag (~0.17) and Ag_2_S (∼2.2), which resulted in a redshift in the plasmonic spectrum, allowing highly sensitive detection of H_2_S.

**Figure 16:**
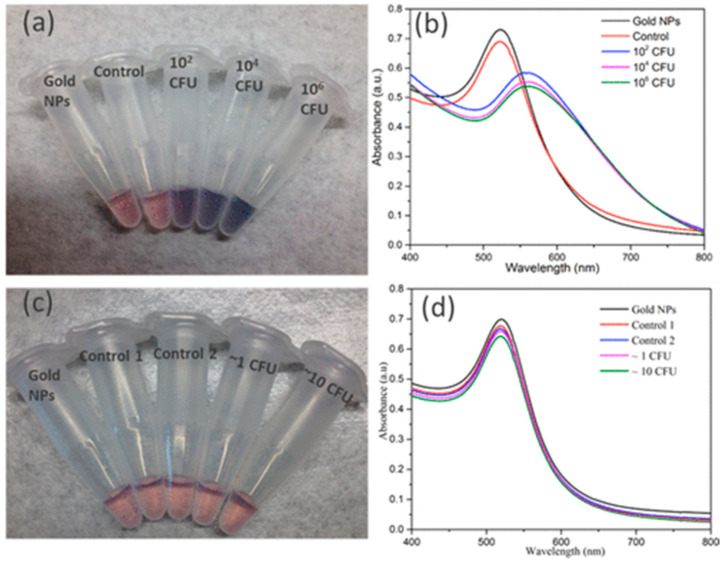
(**a**,**c**) Digital photographs showing detection of *E. coli* using thiolated M13KE and AuNPs. (**b**,**d**) UV–vis spectra. From left to right in (**a**), samples containing AuNPs and: no bacteria or phages (black line in (**b**)), unmodified M13KE with 10^6^ CFU *E. coli* (red line in (**b**)), and thiolated M13KE with *E. coli* at different CFU’s (10^2^, 10^4^, and 10^6^) (blue, magenta, and green lines, respectively, in (**b**)). From left to right in (**c**), samples containing AuNPs and: no bacteria (black line in (**d**)), unmodified M13KE phage and ∼10 CFU of *E. coli* (red and blue lines in (**d**), respectively), and thiolated M13KE phage and ∼10 CFU of *E. coli* (magenta and green lines in (**d**), respectively). Reproduced with permission from [191].

Their biocompatibility and efficient thiol binding ability make gold nanoparticles (AuNPs) an ideal candidate for rapid biosensing based on the sensitivity of the surface plasmon resonance to the aggregation state, which produces a visible color change. Additionally, therefore, antibody-conjugated AuNPs have been used in the rapid colorimetric detection of pathogenic bacterial species in multiple systems, [188,189,190,191] Huan et al. [191] have developed a simple phage display for calorimetric detection of a variety of bacterial species, (Figure 16). This stage involeves Au nanoparticles for simple, efficient and sensitive detection of bacterial cells upto 100 cells with no cross reactivity between different species. 

In addition to gold, silver nanostructures have been employed in plasmonic-based colorimetric sensors [192,193]. Dong et al. [194] demonstrated colorimetric detection of thiosulphate using AgNPs by measuring the variation in RGB value via a smartphone application. Another novel selective colorimetric method for the detection of iodide using citrate-stabilized silver triangular nanoplates was reported by Yang et al. [195]. Researchers have also demonstrated a colorimetric detector using helically structured Ni nanofilms on silicon substrates fabricated via GLAD and found that these films exhibited peroxidase-like activity to catalyze the oxidation of tetramethylbenzidine (TMB) [196]. This colorimetric detection of uric acid was able to achieve a low LOD (3.3 μM) and was found to be sensitive throughout the entire clinically relevant range (15−500 μM).

GLAD-fabricated sculptured thin films have been extensively used for chemical and biosensing, especially for exploiting their LSPR and SERS properties. However, for colorimetric- and wettability-based applications, there are only a few reports are available; therefore, there is a gap yet to be filled. In recent years, considering the huge possibility of tuning the porosity of GLAD-fabricated substrates, there is large scope for surface wetting modification in a wide hydrophilic to superhydrophobic range. Some studies have been carried out on anisotropic wetting and water droplet evaporation on nano-columnar thin films for self-cleaning and the Leidenfrost effect [197,198,199,200]. Wettability-based sensing could be a promising method for the detection of analytes. Gahlaut et al. demonstrated a GLAD nanorod array of silver for H_2_S gas sensing by observing a rapid and drastic change in the water wetting property of the array. As-grown AgNR arrays were found to be hydrophobic with a contact angle of 128°. Exposure to H_2_S gas led to the formation of Ag_2_S on the surface of the AgNR array and resulted in the enhancement of wettability with a water contact angle of 60° [185]. They also presented a novel method of bacterial viability detection using the wetting behavior of the AgNR array and further discriminated antibiotic resistance in bacterial species [38]. The used AgNR array was reutilized in the form of Ag-Ag_2_S nano-heterostructures for various energy and environmental applications, e.g., water purification, hydrogen evolution, and antibacterial activity [201]. All these novel applications of nano-sculptured thin films grown via oblique-angle deposition signify promising future scope in various domains.

### 4.5. Molecular Imaging

The complex biological processes that occur in the cell and its microenvironment can be visualized using a molecular technique. Using this technique, the internal mechanisms of living systems can be visualized. Additionally, certain specific molecules can be studied and the pathways related to the molecules can be unveiled [202]. Disease progression and drug intervention can be effectively studied using imaging. The resolution and noise-to- sound ratio (NSR) is a challenge in molecular imaging. To improve both of these features, the design and development of probes used in imaging in nanoscale regimes have an encouraging future [203]. Contrast materials show unique physical and chemical properties in the nanoscale zone, and this feature is exploited in this molecular imaging. Different chemical and biological methods are used for the fabrication of NPs used in the molecular imaging technique. However, the fabrication cost and the complexity of its preparation has limited its use in biomedical applications. In this current section, we will be discussing the GLAD-based fabrication of NPs and its application in different molecular imaging applications [204,205].

#### 4.5.1. Fluorescence Imaging

Being a very versatile technique, fluorescence finds a wide variety of applications in biomedical fields; these include sensing, bioimaging, diagnosis, and detection [206]. Signal intensity is one of the challenges of fluorescence and it can be met by using metal-enhanced fluorescence (MEF) [207]. MEF is a very powerful and sensitive technique whereby the fluorophores interact with metallic nanoparticles, resulting in an enhancement in fluorescence. Different NP structures affect the plasmonic resonances, which, in turn, enhance fluorescence. The plasmonic effect, along with the enhanced electric field, increases the signal intensity of fluorophores [172,208,209].

Dhruv et al. used a GLAD-fabricated Ag nanorod array to study surface-enhanced fluorescence (SEF), which showed very good enhancement due to the morphology of nanostructures. The spectra of Rh6G were taken on both Ag nanorods and reference substrates. SEF with the length of the nanorods was also found by the group [162]. Jonghyun et al. fabricated Ag nanorods using oblique-angle deposition (OAD) for enhanced fluorescence substrates for improving the sensitivity of microarray analysis used in medical diagnostics. A maximum enhancement of 23 was obtained from the substrates [209].

M.A. Badshah et.al used GLAD-fabricated vertical nanorods for understanding their effect on the sensitivity of DNA microarrays. The group used a kallikrein-related peptidase 7 (KLK7) ssDNA duplex with cyanine 5 dye for checking the sensitivity of the Ag nanorods [165]. Similarly, X Ji et al. used zig-zag Ag nanorod arrays fabricated using GLAD and studied the improved protein and DNA detection. Biotin–neutravidin fluorospheres were attached to the zig-zag Ag nanorods. The enhancement was found to increase with the folding number of the zig-zag nanorods. Additionally, temperature was found to affect the florescence enhancement. Finally, the DNA detection limit was found to be 0.01 pM [210].

#### 4.5.2. Super-Resolution Imaging

On the grounds of medicine, imaging plays a very significant role. Optical microscopy, due to non-invasiveness and simplicity in use, has always been a priority for biologists. Its spatial resolution is a major setback in the case of imaging [211]. Over the years, super-resolution microscopy (SRM) has taken place in imaging techniques to overcome the drawback of optical imaging. Super-resolution microscopy’s higher resolution helps researchers study single molecules and their interactions [212]. Different SRM methods have been developed over the years, such as stochastic optical reconstruction microscopy (STORM), stimulated emitted depletion (STED), photoactivated localization microscopy (PALM), near-field scanning optical microscopy (NSOM), and fluorescence photoactivation localization microscopy (FPLM) [213,214,215]. Along with the high-frequency spatial information, contained in evanescent waves, this type of microscopy uses mathematical modeling and a series of diffraction-limited images, and also optical, electrical setups, to increase the diffraction limit to sublevels. Still, there exist some limitations in super-resolution microscopy; for example, NSOM requires lengthy scan times, whereas FPLM works only with a single-wavelength light source [216,217]. Overall, super-resolution microscopy uses sophisticated instrumentation and is expensive. So, in order to improve the signal-noise-ratio without using a complex setup, different nanostructures are used. Additionally, one of the important applications of super-resolution microscopy is single-molecule imaging; single-molecule imaging is feasible within a limited fluorophore concentration range of 1 pM to 10 nM, whereas protein interaction and enzyme activity require a fluorophore conc. of 1 uM or more. Hence, single-molecule imaging is difficult for biomolecules [218,219,220]. So, in order to tackle this challenge, the density of hotspots should be increased.

Chen et al. used GLAD to fabricate a patterned substrate. To improve the hotspots and enhancement, plasmonic gratings with periodic nanogaps were developed. Silver film growth on polymethylsilsesquioxane (PMSQ) polymer gratings were replicated from HD DVD gratings molds. The silver was deposited at an angle of α = 60° using GLAD. SM fluorescence images were taken on these GLAD-based gratings for a DNA/RNA duplex tagged with cyanine 3 and cyanine 5 labels. GLAD gratings with incorporated nanoprotrusions enabled SM imaging over a wide range of fluorescence from 50 pM to 10 uM. Additionally, the nanoprotrusions allowed the group to use multiple-emitter fitting analysis to solve the crowded field problem created by higher fluorophore concentration. Patchy microspheres fabricated using GLAD setup have also shown potential application in improving the diffraction limit of super-resolution microscopy [221]. Wood et al. performed similar work whereby they used DVD to produce gratings in polymethylsilsesquioxane (PMSSQ) deposited on a glass substrate via microcontact lithography. Ag was deposited using GLAD fabrication. This formed nanoprotrusions and nanogaps that enhanced the LSPR activity and also increased the hotspots, exciting the fluorophores [222].

Shang et al. fabricated microsphere-nanospheres by coating 100 nm thick Ag films using GLAD on the BaTiO3 glass (α = 60°), and the patchy microsphere was made from p-BTG particles. The imaging contrast of p-BTG was found to be improved by a factor of 6.5. Overall, this patchy microsphere improved the imaging contrast without the use of immersion liquid and led to greater advances in the field of super-resolution microscopy.

**Figure 17:**
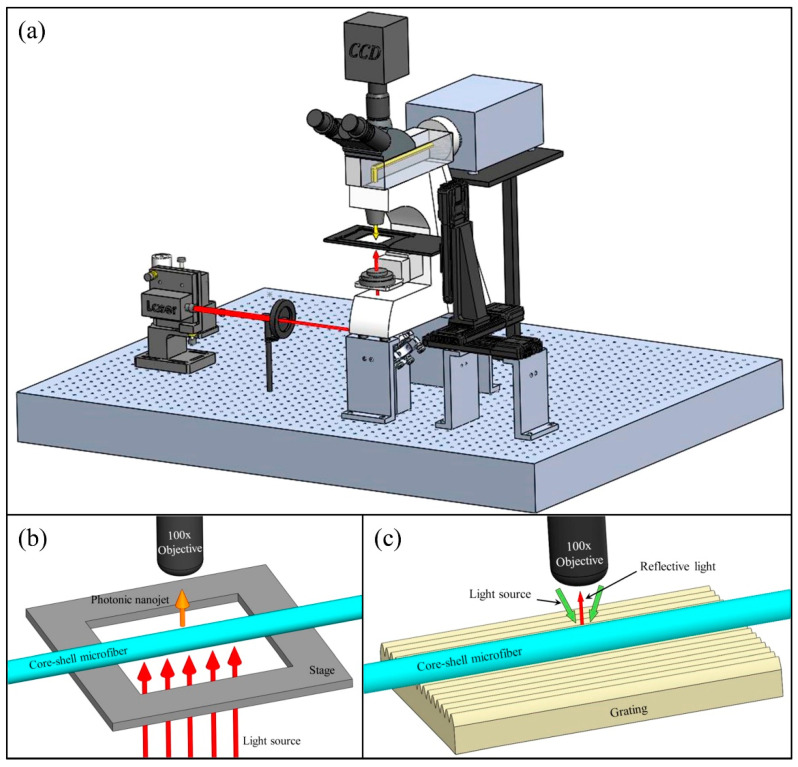
(**a**) Schematic diagram showing SRM microscopy performed. (**b**) Core-shell microfiber shown in the setup whereby the core shell consists of metallic films deposited by GLAD (**c**) Core shell microfiber placed on the top of the specimen. Reproduced with permission from [223].

Liu et al. worked on improving super-resolution optical imaging by using core-shell microfibers. Etching of the optical microfiber was performed to a 6 um diameter followed by the deposition of different metallic thin films using GLAD (Figure 17). By controlling the angle of deposition and substrate orientation, a uniform metallic shell was deposited on the dielectric microfiber core surface. The core-shell microfiber was placed directly on the nanoscale grating surface and SRM was performed. Due to the surface plasmonic properties due to thin metal films, high-definition imaging was obtained compared to the microsphere and microcylinder counterparts. The gold shell produced the best resolution and magnification, with higher contrast [223]. Hence, it was shown by the group that with GLAD-based metallic coating, the intensity of a photonic nanojet can be adjusted and higher-definition imaging is possible.

#### 4.5.3. Photoacoustic Imaging (PA)

A non-invasive biomedical imaging technique exixsts whereby ultrasonic waves are excited into the tissues via irradiation with pulsed or modulated light [224]. The main principle of this method is to convert electromagnetic radiation into acoustic signals through thermoelastic expansion. PA imaging is considered to be one of the prevalent techniques for in vivo studies. This technique plays a crucial role in cancer diagnosis and progression [225], blood oxygen monitoring [226], brain mapping [227], etc. The acoustic signals are detected using an ultrasound receiver which is mostly piezoelectric in nature. Currently, piezoelectric receivers are slowly being replaced by optical receivers to acquire ultrasonic signals, due to their advantage of reduced electrical interconnects [228]. Some of the optical detectors include the Fabry–Perot interferometer (FPI) and micro-ring resonators (MRR) [229]. Hajireza et.al. used an FPI-based detector that utilized nano-thin films fabricated using GLAD. GLAD allows low acoustic impedance of the FP device. These thin films are used for in vivo PA imaging. Multilayers of thin films were deposited using GLAD to act as a mirror in FPI fabrication. TiO_2_ was used as the material due to its high refractive index. An alternate layer of GLAD mirror and parylene C was prepared and a final layer of SiO2 antireflection coating was deposited to improve the optical coupling. This multilayer GLAD optical-resolution PAM(OR-PAM) was used for the demonstration of in vivo imaging of the chorioallantoic membrane (CAM) of a five-day-old chicken embryonic model, showing its application in in vivo imaging [230]. This shows the potential application of GLAD in in vivo PA imaging. The same group also used GLAD-based FPI for measuring carbon fiber networks with a 7 um diameter of each fiber [231].

High resolution PA imaging is possible using multilayer mirrors fabricated using GLAD. This shows the potential of GLAD in improving the resolution of PA microscopy. Additionally, more intense work should be conducted in this field to establish FPI using GLAD and improve its applicability.

### 4.6. In Vivo Application of GLAD

Biomimicking and biomaterial synthesis are new necessary fields in biomedical research. They have an enormous and substantial effect on healthcare, as new materials provide novel properties that can be used in prosthetics and drug carriers [232]. Biomaterials with an anisotropic composition are now currently used in drug delivery for cancer treatment, as biomarkers, as bactericidal agents, for tissue engineering, and for vaccine development [233]. Due to their multi-applicability, anisotropic NPs are currently in demand. They have varied in composition, functionality, shape, and size on the different surfaces of single NPs [234,235]. With this huge diversity, various in vivo applications are possible. Janus particles (JP) and patchy particles, and are the types of NPs used for such applications [236].

JPs or patchy particles are asymmetric colloidal particles with more than one composition and chemical modification at different sites [237]. JPs, due to their multicomposition, can be used for binding with specific molecules and probes [238]. Various methods are used for fabricating JPs, including physical deposition, chemical routes [239], electrochemical methods [240], microfluidics [241], electrohydrodynamic methods, and lithography [242].

**Figure 18:**
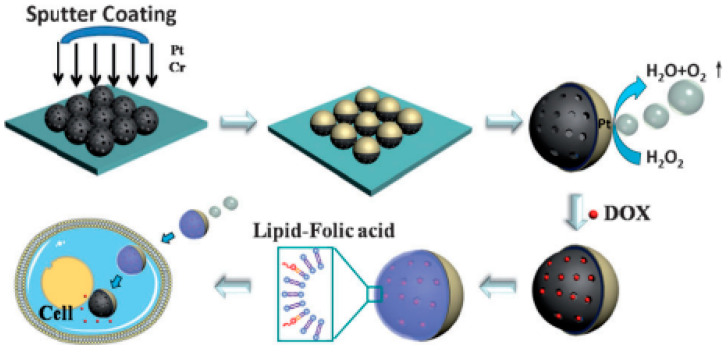
Janus micromotor with one hemisphere coated with Pt and the other hemisphere coated with biotin for specific drug delivery. Reproduced with permission from [245].

As the position of the substrate, as well as the angle, can be varied using the GLAD setup, this vapor flux deposition method is used for obtaining different geometries required in Janus particles [243]. Both the thickness and geometric structures can be controlled by adjusting the flux rate and rotation of the substrate [244]. Xuan et al.’s group designed self-propelling Janus micromotors whereby GLAD was used for depositing Pt at a specific angle over the Si microparticles (Figure 18). Further, the micromotors were modified with biotin so that the charged organic dyes could be transported while maintaining the fast speed of the micromotor. This showed the efficacy of Janus particles in drug delivery applications [245].

Peng et al. combined both top-down and bottom-up fabrication techniques to prepare nanomotors, which were polymeric vesicles deposited with Pt that showed very high drug-loading efficiency. The Janus polymersome nanomotor showed enhanced permeability and retention (EPR) enhancement and also released the encapsulated cargo in a controlled manner under external stimuli [246].

Tejeda-Rodriguez et al. and his group reported making a Janus nanomotor with the capsid from a plant virus on one hemisphere and Pt on the other hemisphere. The Janus viral nanomotor was found to carry and deliver the chemotherapeutic drug tamoxifen to breast tumor cells. The drug release was controlled by a pH-shift mechanism. As the capsid was a biomaterial, it showed an immense advantage over other materials; moreover, surface modification was easy to achieve in this case.

Zhiguang et al.’s group made an extraordinary development in this area by designing micropropellers that can penetrate the delicate vitreous humor of the eye and can perform drug delivery in the retina. The helical magnetic micropropellers were fabricated using GLAD. Ni was deposited onto the Si nanoparticles at an oblique angle. Then, Si was sequentially deposited upon rotation, forming a helical structure. Further, inspired by the sticky liquid layer found on the carnivorous Nepenthes pitcher plant, a non-toxic silicone oil and fluorocarbon coating was used as a slippery surface. Under the influence of a magnetic field, the coated micropropellers showed controlled movement and were able to reach the retina within half an hour [247].

GLAD is a versatile and cheap fabrication method, which has future potential in the fields of drug delivery and biomolecule transportation. Further research could be conducted in the area on its use as a cargo transporter and gene delivery system. GLAD, being a simple and cheap fabrication method, could be explored for various in vivo applications in biological fields. Very little exploration has been done in this field.

### 4.7. Optical and Electrochemical GLAD-Based Sensors

Though GLAD-based biosensors are vastly employed in various sensing systems such as gas sensors [185], optical sensors [6], and electrochemical sensors [248], most of the work that has been conducted and reported employs optical and electrochemical systems due to the high porosity, plasmonic nature, and greater diffusion properties of these metallic nanostructures. Plasmonic metals such as Ag, Au, Cu, and Pt exhibiting high plasmonic resonance in the optical region and the formation of dense electromagnetic hotspots among these nanostructures further increase their sensitivity as optical biosensors. A comparative study highlighting the advantages, LOD, and limitations of GLAD-based optical and electrochemical biosensors is shown below (Table 1).

## 5. Limitations of GLAD Technique

The glancing angle deposition technique possess certain limitations. The nucleation process that occurs is random and cannot be controlled. Due to the shadowing effect taking place, the substrate used for deposition cannot be rough or uneven; otherwise, the portion pointing inside will not be able receive sufficient nucleation due to the shadowing of the outward-pointing portion of the substrate. The technique also involves a lot of material wastage and is not very economical. Due to the high sensitivity and considerable expertise required, limited research has been conducted using this technique. In areas such as molecular imaging, multimodal imaging, and in vivo biosensing, limited research has been conducted using the GLAD technique due to most of these limitations. These gaps can be filled by precise usage and expertise, and the technique can be explored to its fullest potential.

## 6. Conclusions

In conclusion, GLAD is emerging as a convenient technique for the fabrication of nanostructures for different biosensing applications. The homogeneous structures fabricated by GLAD allow reproducible, enhanced, and predictable signals over a large volume. We discussed, in detail, recent advances and strategies in the fabrication techniques of GLAD, their geometrical aspects, and their applications in a number of emerging biochemical and biomedical sensing techniques. In addition, the superiority of the GLAD-based fabrication technique over other conventional techniques and overcoming the limitations of other techniques were discussed in detail. In addition, various types of GLAD -based substrates with improved sensitivity, longevity, and flexibility that have been used in biomedical detection in recent years were discussed. The versatile use of these nanostructures in optical-based biosensing applications such as SPR-, SERS-, and fluorescence-based sensing, as well as colorimetric- and wettability-based detection, has been extensively discussed and studied. The GLAD method in combination with certain new techniques, such as triboelectric methods, new spectroscopic methods, etc., has recently flourished because of their high reproducibility, durability, and sensitivity. The emerging use of GLAD in molecular techniques and in vivo detection was highlighted. A discussion of work conducted by different groups shows the high potential of these fabricated metal nanostructures for biological applications including effective target analyte immobilization. These studies open new horizons for improved bio-detection and applications, and form the basis for further development in the biomedical field, as well as for clinical trials and studies. We also pointed out the challenges of the technology that limit its use in certain applications and emerging fields. Certain limitations of the technique, such as high cost, the need for trained personnel, and sample loss, still need to be addressed. Our review shows the high potential of GLAD-fabricated nanostructures of different morphologies and materials for future biosensing applications. These nanostructures can be a promising tool for certain applications such as single-molecule detection, in vivo biosensing, multimodal imaging, as well as diagnosis, although the use of GLAD-based nanostructures in these fields is yet to be extensively explored.

## Figures and Tables

**Table 1:** Comparative study of GLAD-based optical biosensors and electrochemical biosensors.

GLAD-Based Optical Biosensors	GLAD-Based Electrochemical Biosensors
1.The aspect ratio and morphology of the nanostructures are tuned and optimized to improve sensitivity to a variety of optical properties (fluorescence, absorption, etc.).	1. An electrode surface is coated with biological sensing material for potentiometric, amperometric, or conductimetric measurements [249].
2. Enhanced sensitivity is provided by the plasmonic nature of metals such as Ag, Au, Cu, and Pt.	2. Involves the modulation of electrical properties such as potential, current, or impedance associated with the interaction of biomolecules with the working electrodes [250].
3. By forming electromagnetic hotspots, GLAD-based nanostructures significantly improve optical spectroscopy (Raman, fluorescence, and infrared) and plasmon resonance sensing [24].	3. High porosity, large exposed areas, and excellent diffusion properties make GLAD-based metallic nanostructures excellent electrochemical sensors [251,252].
4. LOD ~ 1 fM [253]	4. LOD ~ 1 µM [251]
5. Nanostructures must be optimized to match optical measurements, must possess high sensitivity, and require trained personnel to operate [54]	5. Comparatively low sensitivity, costly instruments, trained personnel required for their operation [248].

## Data Availability

Not applicable.
